# Identification and mapping of quantitative trait loci for Fusarium head blight resistance in a synthetic hexaploid × hard red spring wheat population

**DOI:** 10.1002/tpg2.70073

**Published:** 2025-07-09

**Authors:** Agnes Szabo‐Hever, Jyoti S. Sharma, Justin D. Faris, Shaobin Zhong, Timothy L. Friesen, Jason D. Fiedler, Andrew J. Green, Guihua Bai, Elias M. Elias, Steven S. Xu

**Affiliations:** ^1^ USDA‐ARS, Cereal Crops Improvement Research Unit, Edward T. Schafer Agricultural Research Center Fargo North Dakota USA; ^2^ Department of Plant Sciences North Dakota State University Fargo North Dakota USA; ^3^ USDA‐ARS, Cereal Disease Laboratory St. Paul Minnesota USA; ^4^ USDA‐ARS, Hard Winter Wheat Genetics Research Unit Manhattan Kansas USA; ^5^ USDA‐ARS, Crop Improvement and Genetics Research Unit, Western Regional Research Center Albany California USA

## Abstract

Fusarium head blight (FHB), caused by *Fusarium graminearum* Schwabe, is one of the most devastating diseases in wheat (*Triticum aestivum* L.). The synthetic hexaploid wheat line Largo was developed from a cross between the durum wheat [*T. turgidum* ssp. *durum* (Desf.) Husn.] variety Langdon and the *Aegilops tauschii* Cosson accession PI 268210, and it was previously found to have a moderate level of FHB resistance. This study was conducted to identify quantitative trait loci (QTL) associated with FHB resistance using a population of 188 recombinant inbred lines (RILs) from a cross between Largo and the susceptible wheat line ND495. The RILs were evaluated for Type II resistance in two greenhouse and two field environments. The disease severity and 90K single‐nucleotide polymorphism marker data were used for QTL analysis, which revealed six QTL on chromosomes 1D, 2D, 5B, and 7D. Four QTL (*QFhb.rwg‐1D*, *QFhb.rwg‐5B*, *QFhb.rwg‐7D.1*, and *QFhb.rwg‐7D.3*) from Largo had minor effects, whereas two QTL (*QFhb.rwg‐2D* and *QFhb.rwg* *‐7D.2*) from ND495 showed large effects on FHB resistance. The result suggested that ND495 may possess suppressor or susceptibility gene(s) suppressing or masking FHB resistance controlled by the resistance QTL. Among these QTL, four coincided with previously reported QTL, including *Fhb9*, and two (*QFhb.rwg‐1D* and *QFhb.rwg‐7D.1*) are likely novel QTL. From the six QTL regions, 10 Kompetitive allele‐specific PCR markers were developed and validated for marker‐assisted selection. The QTL detected from the resistant and susceptible parents enhance our understanding of FHB resistance expression and provide new resources for improving FHB resistance in wheat.

## Core Ideas

- Synthetic wheat line Largo and spring wheat line ND495 are resistant and susceptible to Fusarium head blight (FHB), respectively.
- Quantitative trait loci (QTL) analysis for FHB resistance was performed using an ND495 × Largo population of 188 NIL lines.
- Six FHB‐resistant QTL were identified, with four and two from Largo and ND495, respectively.
- Two QTL from Largo are likely novel, whereas the other four coincided with previously reported QTL.
- Ten Kompetitive allele‐specific PCR markers for the six QTL were developed and validated.

**Abbreviations**
FD: flowering date
FHB: Fusarium head blight
GLM: general linear model
KASP: Kompetitive allele‐specific PCR
LOD: logarithm of odds
LSD: least significant difference
PH: plant height
QTL: quantitative trait loci
RILs: recombinant inbred lines
SHW: synthetic hexaploid wheat
SNP: single‐nucleotide polymorphism
SSR: simple sequence repeat

## 1 INTRODUCTION

Since the early 1990s, Fusarium head blight (FHB), primarily caused by the fungal pathogen *Fusarium graminearum* Schwabe [synonym *Gibberella zeae* (Schw.) Petch], has been one of the most devastating head diseases affecting several cereal crops worldwide, including durum wheat [*Triticum turgidum* L. ssp. *durum* (Desf.) Husn., 2*n* = 4*x* = 28, AABB] and common wheat (*Triticum aestivum* L., 2*n* = 6*x* = 42, AABBDD) (M. Buerstmayr et al., 2020; Chin et al., 2023; Haile et al., 2019). FHB is now ranked as the second most devastating wheat disease in the world after leaf rust (caused by *Puccinia triticina* Eriks), causing 2.85% yield loss annually, value at $5.6 billion globally (Savary et al., 2019; Wulff & Jones, 2020). In addition, the fungus produces mycotoxins, which contaminate wheat grain and threaten human and animal health (Bottalico & Perrone, 2002; Pestka, 2010). To mitigate the threat from FHB outbreaks, there has been a constant need to develop resistant wheat varieties in wheat breeding programs worldwide.

Compared to other common fungal diseases such as leaf rust or stem rust (caused by *P. graminis* Pers. f. sp. *tritici* Eriks and E. Henn), breeding for FHB resistance is difficult due to its complexity. An innate challenge is that the expression of FHB resistance in plants is highly affected by the environment and genotype × environment interactions (Miedaner et al., 2001). Warm temperature and high humidity during flowering, when the plant is in the most sensitive development stage for fungal infection, cause a high FHB severity (Andersen, 1948; Parry et al., 1995) even on resistant genotypes. On the contrary, dry weather conditions during flowering, a critical period for disease development, often result in low FHB severity on susceptible genotypes even in mist‐irrigated nurseries (Szabo‐Hever et al., 2018). Furthermore, several morphological and agronomic traits, such as plant height (PH), spike morphology, and flowering date (FD), can often affect fungal infection and/or FHB disease development (H. Buerstmayr et al., 2009; Lu et al., 2013; Mesterhazy, 1995), further complicating the selection of resistant genotypes in breeding.

Another challenge for wheat breeders is that FHB resistance is a complex trait, usually controlled by multiple quantitative trait loci (QTL). In the past two decades, >600 FHB resistance QTL across all 21 wheat chromosomes have been reported (see review by H. Buerstmayr et al., 2009; M. Buerstmayr et al., 2020; Venske et al., 2019). However, only nine of the QTL have been formally designated as *Fhb1* through *Fhb9* (review by Viviani et al., 2025). Most of the other reported QTL had minor or moderate effects, often highly affected by environmental factors and genetic backgrounds. Many of the FHB resistance QTL overlap with the QTL associated with morphological traits, which often mask true resistance and thus limit the efficient utilization of FHB resistance QTL in wheat breeding (H. Buerstmayr et al., 2009; M. Buerstmayr et al., 2020; Lu et al., 2013). So far, *Fhb1* on chromosome 3B derived from wheat cultivar Sumai 3 has been verified to show a major effect on FHB resistance consistently, and it has been effectively used in wheat breeding programs around the world (H. Wang et al., 2020). As a result, many wheat cultivars carrying *Fhb1* have been developed and extensively used in production in regions with frequent FHB outbreaks since 1999 (M. Buerstmayr et al., 2020; Hao et al., 2020).

The widespread deployment of *Fhb1* in modern wheat cultivars worldwide can reduce losses caused by FHB. However, the large‐scale utilization of wheat cultivars with *Fhb1* in breeding and wheat production may pose a potential risk of FHB epidemics due to pathogen adaptation and the narrow diversity of genes for FHB resistance. To widen the genetic sources of FHB resistance for breeding, wheat germplasms with novel resistance QTL need to be identified. Hexaploid wheat originated from the natural hybridization between tetraploid wheat (*T*. *turgidum*) and the D genome progenitor *Aegilops tauschii* Coss. (2*n* = 2*x* = 14, DD) (Cox, 1997; Lev‐Yadun et al., 2000; Marcussen et al., 2014; Salamini et al., 2002). Because only limited tetraploid wheat or *Ae. tauschii* genotypes participated in the origin of hexaploid wheat, a narrow genetic diversity has been incorporated into modern hexaploid wheat germplasm (Gaurav et al., 2022; L. Zhang et al., 2008). Although tetraploid wheat is generally more susceptible to FHB than hexaploid wheat, several resistant accessions and QTL have been identified in wild and cultivated emmer wheat (*T. turgidum* subsp. *dicoccoides* and *T. turgidum* subsp. *dicoccum*) (M. Buerstmayr et al., 2012; Oliver et al., 2008; Ruan et al., 2012; Q. Zhang et al., 2014), Persian wheat (*T. turgidum* subsp. *carthlicum*), and durum wheat (H. Buerstmayr et al., 2003; M. Buerstmayr et al., 2013, 2012; Gladysz et al., 2007; Kumar et al., 2007; Oliver et al., 2008, 2007; Otto et al., 2002; Somers et al., 2006; Q. Zhang et al., 2014). Compared to tetraploid wheat, *Ae. tauschii* is rarely evaluated for FHB resistance due to its wild, grassy characteristics. However, Brisco et al. (2017) evaluated 109 *Ae*. *tauschii* accessions for reactions to FHB and identified several resistant accessions.

Tetraploid wheat and *Ae*. *tauschii* accessions with FHB resistance have not been fully utilized to improve common wheat germplasm. One effective method of incorporating FHB resistance genes from these species into common wheat could be developing synthetic hexaploid wheat (SHW, × *Aegilotriticum* spp., 2*n* = 6*x* = 42, AABBDD). Since the 1990s, numerous SHW lines have been created through crosses between various tetraploid wheat and *Ae. tauschii* accessions (Mujeeb‐Kazi et al., 2001; Ogbonnaya et al., 2013; Szabo‐Hever et al., 2018; Xu et al., 2010). Reactions to FHB in SHW germplasm were previously investigated in several studies, resulting in the identification of multiple resistant lines (Mujeeb‐Kazi et al., 2001; Oliver et al., 2005; Szabo‐Hever et al., 2018). Szabo‐Hever et al. (2018) reported 13 SHW lines with a high level of FHB resistance. These SHW lines may be useful for incorporating FHB resistance genes from tetraploid wheat and *Ae. tauschii* into common wheat.

Among the FHB‐resistant SHW lines identified by Szabo‐Hever et al. (2018), one line derived from a cross between the durum variety Langdon and the *Ae*. *tauschii* accession PI 268210 was previously named Largo and released as wheat germplasm carrying gene *Gb3* for resistance to greenbug [*Schizaphis graminum* (Rondani)] (Joppa & Williams, 1982). In addition to its resistance to greenbug, Largo also exhibits resistance to multiple diseases, including stem rust, FHB, tan spot [caused by *Pyrenophora tritici‐repentis* (Died.) Drechsler], septoria nodorum blotch [caused by *Parastagonospora nodorum* (Berkeley) Quaedvlieg, Verkley & Crous], and septoria tritici blotch [caused by *Mycosphaerella graminicola* (Fuckel) J. Schröt.] (Adhikari et al., 2015; Friesen et al., 2008; Sharma et al., 2021; Szabo‐Hever et al., 2018). A mapping population of 226 recombinant inbred lines (RILs) was previously developed from the cross between hard red spring wheat line ND495 and Largo (Sharma et al., 2021). In this study, we aimed to identify the FHB resistance QTL in the RIL population of Largo × ND495 and develop and validate user‐friendly markers for marker‐assisted selection.

**Core Ideas**

- Synthetic wheat line Largo and spring wheat line ND495 are resistant and susceptible to Fusarium head blight (FHB), respectively.
- Quantitative trait loci (QTL) analysis for FHB resistance was performed using an ND495 × Largo population of 188 NIL lines.
- Six FHB‐resistant QTL were identified, with four and two from Largo and ND495, respectively.
- Two QTL from Largo are likely novel, whereas the other four coincided with previously reported QTL.
- Ten Kompetitive allele‐specific PCR markers for the six QTL were developed and validated.

## 2 MATERIALS AND METHODS

### 2.1 Plant materials

Largo (CI 17895) is an SHW line developed by Dr. L. R. Joppa through a cross between durum wheat Langdon and *Ae. tauschii* PI 268210 (Joppa & Williams, 1982). ND495 (pedigree: Justin*2/3/ND 259/Conley//ND 112) was developed at North Dakota State University, Fargo, ND, and is susceptible to FHB. To identify and map resistance genes in Largo, we developed a population of 226 RILs by crossing ND495 with Largo (Sharma et al., 2021). For this study, we randomly selected 188 RILs to identify and map FHB resistance QTL. The common wheat varieties Sumai 3 and Wheaton were used as the resistant and susceptible checks, respectively, in the FHB evaluation in the greenhouse and field nurseries. A set of 25 SHW lines with moderate and high levels of FHB resistance reported by Szabo‐Hever et al. (2018) was used to validate the new markers linked to the QTL identified in this study.

### 2.2 Evaluation of FHB resistance

The Type II resistance (resistance to spread in the spike) of the ND495/Largo RIL population, its parental lines, and checks (Sumai 3 and Wheaton) was assessed in two greenhouse seasons and two field environments, using the methods and procedures previously described in Chu et al. (2011), Q. Zhang et al. (2014), and Szabo‐Hever et al. (2018). A randomized complete block design with three replicates was used for all experiments.

Greenhouse experiments were conducted in winter 2018 (18GH) and spring 2019 (19GH). Three seeds from each genotype were planted in a 6‐inch clay pot per replicate, and thus, the genotypes were represented by a total of nine plants per experiment. The temperature was maintained at 22°C–25°C in the greenhouse, which was also supplemented with artificial light for a 16‐h photoperiod. The inoculum was prepared at a concentration of 50,000 spores mL^−1^ from three strains of pathogenic *F*. *graminearum* (Szabo‐Hever et al., 2018). Inoculation was performed according to the point inoculation method described by Stack et al. (2002), wherein 10 µL of inoculum was injected into the central spikelet located in the middle of each spike at anthesis. In each pot, 8–12 spikes were inoculated. Each inoculated spike was misted using a hand sprayer and then covered with a misted 5‐inch plastic bag for 72 h (Szabo‐Hever et al., 2018). Disease severity was determined by counting the number of diseased spikelets and total spikelets on each spike at 19 days post‐inoculation, and the scores were calculated as the percentage of diseased spikelets per spike.

In the field experiments, the plant materials were planted in hill plots with 15 seeds per hill and a seeding spacing of 25 × 25 cm in a mist‐irrigated FHB nursery located at Fargo, North Dakota. The evaluations were performed using spawn inoculation in 2018 (18F) and 2019 (19F). Planting, inoculum preparation, and inoculation were performed as described by Chu et al. (2011) and Szabo‐Hever et al. (2018). The inoculum used in the experiments was prepared by inoculating autoclaved corn seeds with a mixture of spores from 20 different *F. graminearum* strains. As reported by Puri and Zhong (2010), the 20 strains used in the study were collected from the field in North Dakota and consisted of 10 producers of 3‐acetyl‐deoxynivalenol and 10 producers of 15‐acetyl‐deoxynivalenol. During the boot stage of the earliest lines, the *Fusarium*‐spoiled corn inoculum was evenly applied to all the plots at a rate of 35.6 g m^−2^. Subsequently, the nursery was misted at 1‐h intervals for 2 min for 12 h per day (4:00 p.m. to 4:00 a.m.), until approximately 14 days after the inoculation of the latest flowering genotypes. The disease severity was recorded 19 days post anthesis as the percentage of infected spikelets on each head estimated based on the visual scale of 10 categories of infection (1 = 0, 2 = 7%, 3 = 14%, 4 = 21%, 5 = 33%, 6 = 50%, 7 = 66%, 8 = 75%, 9 = 90%, and 10 = 100%) described by Stack and McMullen (1998). The disease severity in each plot was calculated by computing the average severity of all heads, based on 10 scored spikes.

PH and FD data were collected in both field experiments (PH18 and PH19), and overall averages for PH (PHALL) and FD (FDALL) were calculated. The measurement of PH was taken from the surface of the ground to the highest point of the spike while excluding the awns. The FD was calculated starting from June 11 in 2018 and from June 25 in 2019, when about 50% of spikes in a hill were flowering. Both PH and FD data were used to determine the correlation of these traits with FHB disease severity.

### 2.3 Statistical analyses

The software JMP Genomics 7 (SAS Institute) was utilized to compute descriptive statistics and to conduct a normality test for the distribution of disease severity, FD, and PH, using the Goodness of Fit option with the Shapiro–Wilk test as described by Szabo‐Hever et al. (2018). To examine the homogeneity of variances for disease severity, FD, and PH among the experiments, Levene's test was employed for data sets that exhibited a non‐normal distribution. In contrast, Bartlett's test was utilized for data sets with normal distribution. The tests were conducted under the general linear model (GLM) procedure using SAS Program version 9.4 (SAS Institute). The least significant difference (LSD) values were calculated using the GLM procedure. The correlation coefficients between disease severity and PH, as well as disease severity and FD, were computed using the PROC CORR procedure (SAS Institute). The broad sense heritability across environments was estimated in accordance with Nyquist and Baker (1991) by applying the following formula: *H* ^2^ across environments = 1 − (MS_G×E_/MS_G_), where MS_G×E_ and MS_G_ represent mean square genotype × environment and mean square genotype, respectively.

### 2.4 Marker data and QTL analysis

A population of 188 RILs was genotyped with Illumina 90K iSelect genotyping assay using Illumina's iSelect method following the manufacturer's protocols for processing and clustering (Illumina Inc.) (Sharma et al., 2021). A total of 11,425 segregating single‐nucleotide polymorphism (SNP) markers were detected in the RIL population. In addition, 22 simple sequence repeat (SSR) markers were polymorphic between ND495 and Largo and segregated in the RIL population. Linkage analysis was conducted using the computer program MapDisto 2.1.7 (Heffelfinger et al., 2017). A set of 11,447 markers was grouped using the command “Find linkage groups” with a logarithm of odds (LOD) > 3.0 and a maximum theta of 0.30. The markers were further filtered by deleting redundant co‐segregating markers and markers with a missing data rate of 10% or higher. To determine the best order of each group, the “Order a linkage group”, “Ripple order”, “Check inversions”, and “Drop locus” commands were used. Markers with segregation distortion were filtered out. Linkage distances were calculated using the Kosambi mapping function, as described by Kosambi (1943). The number of markers in the whole genome linkage map (Table S1) was reduced by leaving only one representative marker from each unique locus. Error candidates and missing data were restored using the commands “Replace error candidates by flanking genotype” and “Replace missing data by flanking genotype,” respectively. The final linkage map, consisting of 1698 markers, was used to conduct QTL analysis and prepare figures (Table S2).

QTL analysis was performed using the method described by Q. Zhang et al. (2014). Composite interval mapping was conducted using the software program QGene v.4.4.0 (Joehanes & Nelson, 2008). A 1000‐iteration permutation test was carried out, which determined a critical threshold of 3.3 at the 0.05 probability level. The *R* ^2^ and *Add effect* functions in the program were used to calculate the percentage of phenotypic variance explained by a QTL and its additive effect, respectively. QTLNetwork 2.1 program (J. Yang et al., 2005) was used to explore QTL × QTL interaction using multiple QTL mapping function.

To compare the differences in FHB resistance among different QTL haplotypes, the RILs with no missing allele information at the significant markers were grouped based on QTL alleles. The mean differences in the FHB severities among the QTL haplotype groups were tested using the LSD procedure PROC GLM performed in SAS Program version 9.4 (SAS Institute). Box plots were created in JMP Genomics 7 (SAS Institute) using the *Graph Builder* function to graphically demonstrate the difference among QTL groups.

### 2.5 Development and validation of Kompetitive allele‐specific PCR (KASP) markers linked to identified QTL

The significant 9K and 90K SNP markers at or near the peak regions of the identified FHB resistance QTL were converted to KASP markers (Table 1). The primer sequences of the KASP markers were obtained from the PolyMarker database (https://www.polymarker.info/designed_primers). KASP markers were validated by genotyping the ND495/Largo population and a set of 25 SHW wheat lines showing a moderate or high level of FHB resistance (Szabo‐Hever et al., 2018) using the assay protocol as described in Nirmala et al. (2017) in the USDA‐ARS Small Grain Genotyping Laboratory, Fargo, ND.

**TABLE 1:** Kompetitive allele‐specific PCR (KASP) markers designed using the significant single‐nucleotide polymorphisms (SNPs) in the quantitative trait loci (QTL) regions mapped in the population of the ND495/Largo recombinant inbred lines (RILs).

KASP marker^a^	SNP position (cM)^b^	OTL	Primer type	Primer sequence
*KASP‐IWB17549‐1D*	45.4	*QFhb.rwg‐1D*	A1	TCATCTATTTTATGGGCAGGTACAA
			A2	TCATCTATTTTATGGGCAGGTACAC
			C	GAGGAGGAGCACTTGGAACA
*KASP‐IWB6869‐1D*	48.6	*QFhb.rwg‐1D*	A1	GAGCTCTTCTCTGCCTTCCT
			A2	GAGCTCTTCTCTGCCTTCCG
			C	CGAGCTTGAGAGTATCAGTGAA
*KASP‐IWB57869‐2D*	72.7	*QFhb.rwg‐2D*	A1	ACAAAGCTGACAATTCCACCA
			A2	ACAAAGCTGACAATTCCACCG
			C	ACAAGCAAATAGTAAGGCATAAACA
*KASP‐IWA4789‐2D*	75.2	*QFhb.rwg‐2D*	A1	AGCTCCGTGCAGTGCTTT
			A2	AGCTCCGTGCAGTGCTTC
			C	GTACCATTCTCTACGGCACC
*KASP‐IWB484‐2D*	82.4	*QFhb.rwg‐2D*	A1	TTTCAGATACTTCAGGGCTTCA
			A2	TTTCAGATACTTCAGGGCTTCG
			C	GATTGACGGGGTGGGTACA
*KASP‐IWB52117‐5B*	77.8	*QFhb.rwg‐5B*	A1	GGAATTACCAGGGAGACAGGA
			A2	GGAATTACCAGGGAGACAGGC
			C	TGTCCTGGTTCATTGTAGACTTC
*KASP‐IWB15280‐7D*	42.2	*QFhb.rwg‐7D.1*	A1	ATTCTTCATCAATAGTGTCCCCA
			A2	ATTCTTCATCAATAGTGTCCCCG
			C	TGCACCGGCGCCTAATTTT
*KASP‐IWA8486‐7D*	64.3	*QFhb.rwg‐7D.2*	A1	CAGATGTACCCTCCAATGCAT
			A2	CAGATGTACCCTCCAATGCAC
			C	TAGCCATGGGTGGGACATAT
*KASP‐IWB14320‐7D*	71.1	*QFhb.rwg‐7D.2*	A1	CCAACCAATCTCAAGCTATCTTCA
			A2	CCAACCAATCTCAAGCTATCTTCG
			C	TCTCAGGTTGACTTGGCACC
*KASP‐IWB8024‐7D*	88.2	*QFhb.rwg‐7D.3*	A1	TGGCTCCTCAGTTTGAAATGT
			A2	TGGCTCCTCAGTTTGAAATGC
			C	AATCTATCAAGGAGAAGAGCACA

*Note*: Primer types: A1 (FAM) and A2 (HEX) are allele‐specific primers, and C is common primer.

^a^ KASP markers are named using a formula “KASP‐9K or 90K SNP name‐Chromosome.”

^b^ SNP position based on the ND495/Largo RIL population linkage map.

## 3 RESULTS

### 3.1 Evaluation of ND495/largo population for resistance to FHB

**FIGURE 1:**
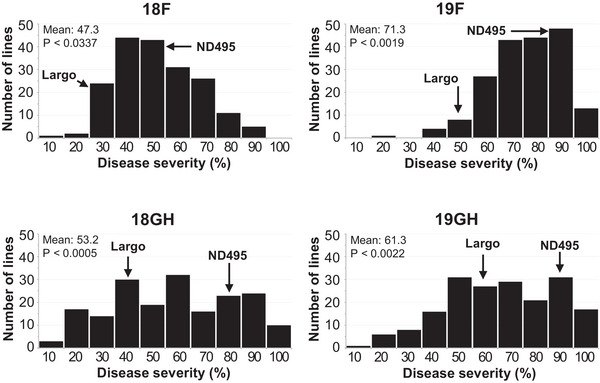
Distribution of Fusarium head blight (FHB) disease severities among the recombinant inbred lines (RILs) of the ND495 × Largo population in the field and greenhouse experiments conducted in 2018 and 2019 (18F, 19F, 18GH, and 19GH). Letter “P” represents probability from normality test for the distribution of disease severity.

The 188 RILs, their parents (ND495 and Largo), and two checks (Sumai 3 and Wheaton) were evaluated for FHB resistance in two greenhouse experiments (Tables S3 and S4) and two field experiments (Tables S5 and S6). However, two lines (NL9 and NL130) were not evaluated due to late heading. The Levene's test for FHB severity showed heterogeneity of error variances across two field and two greenhouse experiments (*P* < 0.0001, *df* = 3). However, the data within experiments showed homogeneity (18F: *p* = 0.2383, *df* = 2; 19F: *p* = 0.4156, *df* = 2; 18GH: *p* = 0.3436, *df* = 2; 19GH: *p* = 0.1655, *df* = 2). The evaluation data showed that the individual genotypes in the tested plant materials had variable expressions of FHB resistance in the experiments (Figure 1). The highest disease pressure was observed in the field in 2019 due to the warm and humid weather after plant heading. The resistant and susceptible checks had the expected level of FHB resistance in all environments (Sumai 3: 3.0%–17.5%, and Wheaton: 69.0%–94.2%; Table 2). Largo (23.3%–52.3%) exhibited lower FHB severities than ND495 (40.0%–89.2%) across all four experiments, with the differences reaching statistical significance (*p* < 0.05) in three of the experiments: 19F, 18GH, and 19GH (Table 2; Figure 1). The heritability (*H* ^2^) values for FHB severity were high (0.72, 0.89, and 0.75) for field experiments, greenhouse experiments, and combined data from all four experiments, respectively (Table 2), indicating good reproducibility of FHB severity data among the experiments.

**TABLE 2:** Descriptive statistics on Fusarium head blight (FHB) severity, plant height, and flowering date for the ND495/Largo recombinant inbred lines (RILs), their parents, and checks (Sumai 3 and Wheaton) evaluated in the field and greenhouse experiments.

Data set^a^	*df*	Mean	SD	Median	Range	ND495	Largo	Sumai 3	Wheaton	LSD	*H* ^2^
**FHB severity (%)**											
18F	2	47.3	15.8	46.2	3.0–87.5	40.0	23.3	3.0	69.0	24.8	
19F	2	71.3	14.5	72.0	17.5–97.2	89.2	44.3	17.5	94.2	17.5	
FHBF	1	59.4	13.3	59.4	10.3–91.5	64.6	33.8	10.3	81.6	15.1	0.72
18GH	2	53.2	24.2	54.3	6.2–100.0	78.9	38.6	6.2	90.6	24.6	
19GH	2	61.3	21.8	62.0	4.5–100.0	87.6	52.3	4.5	81.3	20.4	
FHBGH	1	57.1	21.9	56.5	5.3–100.0	83.3	45.5	5.3	86.0	16.6	0.89
FHBALL	3	58.1	15.0	57.6	7.8–93.7	74.0	39.6	7.8	83.8	11.7	0.75
**Plant height (cm)**											
PH18	2	85.8	10.1	86.0	57.7–111.7	66.0	100.0	87.3	65.0	8.1	
PH19	2	89.7	10.7	88.3	60.0–120.0	70.0	110.0	78.3	70.0	7.5	
PHALL	1	87.9	10.2	87.5	58.8–120.0	68.0	105.0	82.8	67.5	5.6	0.94
**Flowering date (day)**											
FD18	2	23.4	6.6	22	13–41	14	36	23	18	6.9	
FD19	2	17.8	5.9	16	11–41	13	34	14	15	4.6	
FDALL	1	20.6	6.0	19	12–41	14	35	19	16	4.1	0.89

Abbreviations: *H* ^2^, broad‐sense heritability; LSD, least significant differences (*α* < 0.05); SD, standard deviation.

^a^ Data set: 18F and 19F are FHB severity data from field experiments in 2018 and 2019, respectively; 18GH and 19GH are FHB severity data from greenhouse experiments in 2018 and 2019, respectively; FHBF and FHBGH are combined FHB severity data from field and greenhouse data, respectively. FHBALL is combined FHB severity data of all FHB experiments (18F, 19F, 18GH, and 19GH); PH18 and PH19 are plant height data from field experiments in 2018 and 2019, respectively, PHALL is combined plant height data of the field experiments in 2018 and 2019, respectively; FD18 and FD19 are flowering date data from field experiments calculated starting from June 11 in 2018 and June 25 in 2019, respectively; FDALL is combined flowering date data from the field experiments in 2018 and 2019.

### 3.2 Effects of PH and days to flowering on FHB severity in the field experiments

Bartlett's test for PH showed homogeneity across the two field seasons and within experiments as well (PH18: *χ* ^2^_df = 2_ = 0.79, *p* = 0.6742; PH19: *χ* ^2^_df = 2_ = 0.97, *p* = 0.6170; PHALL: *χ* ^2^_df = 1_ = 1.81, *p* = 0.1786). PH showed a wide variation among RILs, ranging from 58.8 to 120.0 cm (Table 2). Levene's test indicated significant heterogeneity in FD data both among experiments (*p* < 0.0001) and within the FD19 experiment (*p* = 0.0038), whereas the data within FD18 were homogeneous (*p* = 0.3339). In FD18, Pearson's correlation coefficients between FHB severity and PH or FD were −0.012 and 0.089, respectively, and were not statistically significant (*p* = 0.8756 and *p* = 0.2239, respectively; Table 3). In FD19, weak but statistically significant negative correlations were observed between FHB severity and PH (*r* = −0.198; *p* = 0.0065), as well as between FHB severity and FD (*r* = −0.250; *p* = 0.0006). These results indicate that PH and FD did not significantly affect FHB severity in 2018, whereas in 2019 shorter plants and/or early flowering plants had higher disease severity.

**TABLE 3:** Pair‐wise correlation coefficients between Fusarium head blight (FHB) severity, flowering date, and plant height data.

Data set^a^	FD18	FD19	PH18	PH19	18F	19F	18GH
FD19	0.825***						
PH18	0.536***	0.435***					
PH19	0.496***	0.463***	0.874***				
18F	0.089	0.058	−0.012	−0.009			
19F	−0.162*	−0.250***	−0.227**	−0.198**	0.553***		
18GH	−0.382***	−0.361***	−0.173*	−0.199**	0.242***	0.346***	
19GH	−0.197**	−0.187*	0.020	−0.002	0.300***	0.384***	0.802***

^a^ Data set: 18F and 19F are FHB severity data from field experiments in 2018 and 2019, respectively; 18GH and 19GH are FHB severity data from greenhouse experiments in 2018 and 2019, respectively. PH18 and PH19 are plant height data from field experiments in 2018 and 2019, respectively; FD18 and FD19 are flowering date data from field experiments in 2018 and 2019, respectively.

*, **, and *** denote significance at the 0.05, 0.01, and 0.001 probability levels.

### 3.3 Linkage groups and QTL analysis

A total of 1682 SNP and 16 SSR markers that segregated among the RILs were used to construct a genetic linkage map, covering a genetic length of 2406.1 cM with an average marker density of 1.4 cM per marker. Maps for the A, B, and D genomes spanned 845.7, 706.5, and 853.9 cM with an average density of 1.4, 1.1, and 1.8 cM per marker, respectively (Table S7). Chromosome 4D was the shortest (69.1 cM), and chromosome 5A was the longest (156.4 cM) in the linkage map. The number of markers per chromosome varied from 21 (4D) to 160 (5A), and marker densities varied from 0.87 cM per marker on chromosome 7B to 3.29 cM per marker on chromosome 4D.

**FIGURE 2:**
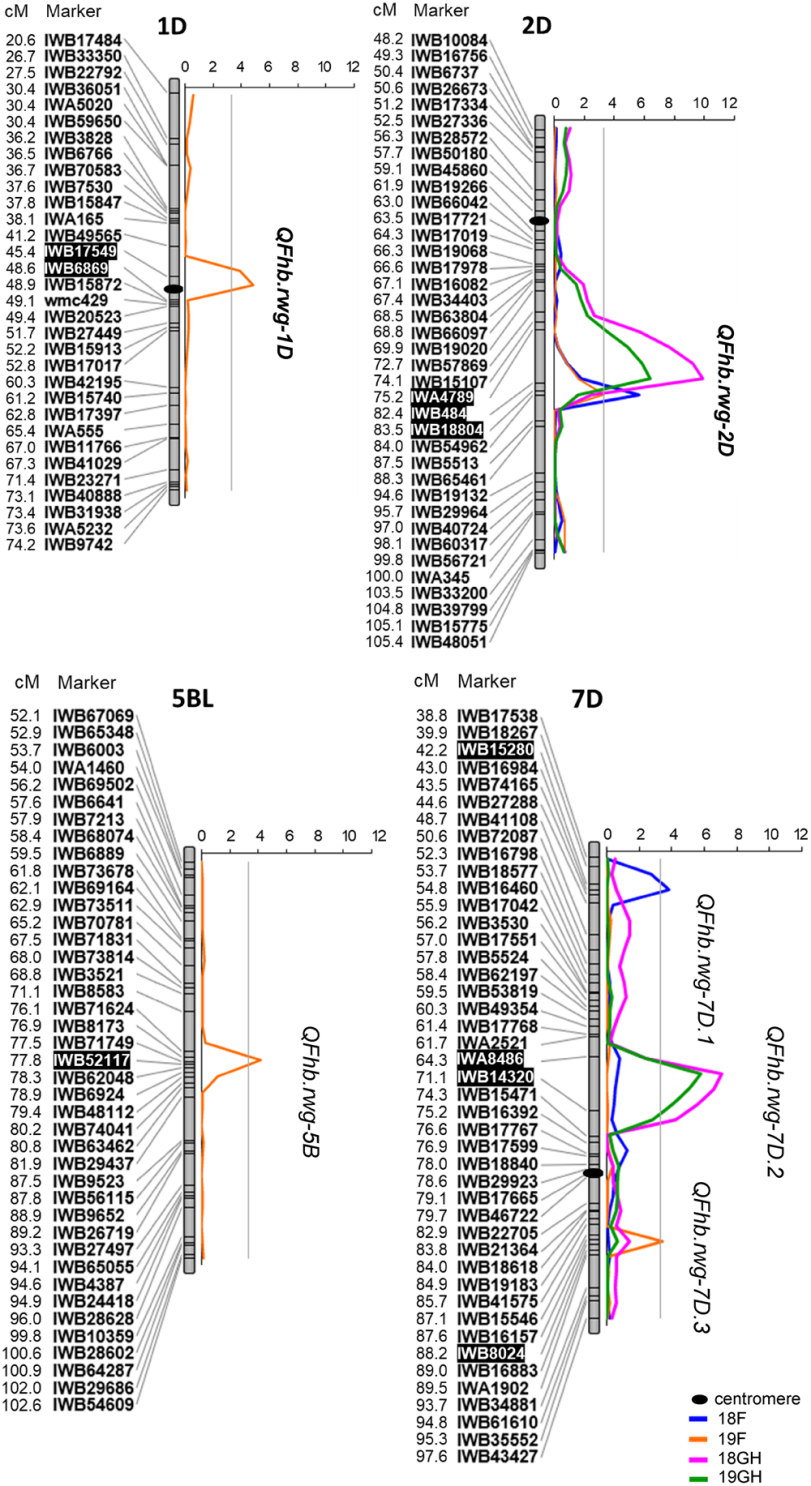
Logarithm of odds (LOD) curves of Fusarium head blight (FHB) resistance quantitative trait loci (QTL) in the ND495 × Largo population. The names of marker loci and their positions in centiMorgans (cM) are shown to the left of the linkage maps. The vertical solid gray line indicates the LOD significance threshold of 3.3. *R* ^2^ and LOD values of the QTL are shown in Table 4. Blue and orange solid lines indicate field experiments conducted in 2018 and 2019, shown as data of 18F and 19F, respectively. The pink and green solid lines indicate greenhouse experiments in 2018 and 2019, shown as 18GH and 19GH, respectively. Black‐filled ellipsis indicates the position of the centromere on chromosomes.

The homogeneity tests showed that the error variances across the two field and two greenhouse experiments were not homogenous. Therefore, the FHB severity data from the four environments (18F, 19F, 18GH, and 19GH) were analyzed separately for QTL detection. A total of six QTL were associated with FHB resistance (Figure 2; Table 4). Among these QTL, three were located on chromosomes 1D, 2D, and 5B and designated *QFhb.rwg‐1D*, *QFhb.rwg‐2D*, and *QFhb.rwg‐5B*, respectively. Three other QTL were all located on chromosome 7D, and they are designated as *QFhb.rwg‐7D.1*, *QFhb.rwg‐7D.2*, and *QFhb.rwg‐7D.3*, respectively. Among these QTL, *QFhb.rwg‐2D*, which was derived from the susceptible parent ND495, was the only QTL detected in all four experiments (18F, 19F, 18GH, and 19GH). *QFhb.rwg‐2D* had the largest additive effect (−6.99, −4.48, −11.12, and −8.17, respectively), with LOD values of 5.64, 3.32, 9.93, and 6.39, and it explained 13%, 8%, 22%, and 15% of the phenotypic variation in 18F, 19F, 18GH, and 19GH, respectively (Figure 2; Table 4). With 18GH and 19GH data, *QFhb.rwg‐2D* peaked in a 7.2‐cM chromosome interval between *IWA4789* and *IWB484* at the physical position between 75.2 and 82.4 cM (481.6 and 533.1 Mb). With the 18F and 19F data, this QTL showed the highest LOD value at marker *IWB18804* at position 83.5 cM (538.7 Mb).

**TABLE 4:** Quantitative trait loci (QTL) in the ND495/largo recombinant inbred line population associated with Fusarium head blight (FHB) resistance.

			Peak or significant markers			Flanking markers				
Chr.	QTL	Data set^a^	Marker name	Position (cM)	Position (Mb)^b^	Marker interval	Genetic interval (cM)	Add^c^	LOD	*R* ^2^
1D	*QFhb.rwg‐1D*	19F	IWB17549	45.4	n.d.	IWB49565–IWB15872	41.2–48.9	4.02	4.82	0.11
			IWB6869	48.6	250.1					
2D	*QFhb.rwg‐2D*	18GH	IWA4789	75.2	481.6	IWB15107–IWB18804	74.1–83.5	−11.12	9.93	0.22
			IWB484	82.4	533.1					
		19GH	IWA4789	75.2	481.6	IWB15107–IWB18804	74.1–83.5	−8.17	6.39	0.15
			IWB484	82.4	533.1					
		18F	IWB18804	83.5	538.7	IWB484–IWB54962	82.4–84.0	−6.99	5.64	0.13
		19F	IWB18804	83.5	538.7	IWB484–IWB54962	82.4–84.0	−4.48	3.32	0.08
5B	*QFhb.rwg‐5B*	19F	IWB52117	77.8	668.5	IWB71749–IWB62048	77.5–78.3	3.7	4.16	0.1
7D	*QFhb.rwg‐7D.1*	18F	IWB15280	42.2	42.5	IWB18267–IWB16984	39.9–43.0	4.13	3.83	0.09
	*QFhb.rwg‐7D.2*	18GH	IWA8486	64.3	117.8	IWA2521–IWB15741	61.7–74.3	−9.21	7.11	0.16
			IWB14320	71.7	146.1					
		19GH	IWA8486	64.3	117.8	IWA2521–IWB15741	61.7–74.3	−7.74	5.79	0.14
			IWB14320	71.7	146.1					
	*QFhb.rwg‐7D.3*	19F	IWB8024	88.2	516.0	IWB16157–IWB16883	87.6–89.0	3.2	3.39	0.08

Abbreviations: Chr, chromosome; LOD, logarithm of the odds; n.d., data not available.

^a^ Data set: 18F and 19F are FHB severity data from field experiments in 2018 and 2019, respectively; 18GH and 19GH are FHB severity data from greenhouse experiments in 2018 and 2019, respectively.

^b^ Data based on the Chinese Spring reference genome v1.0 (IWGSC, 2018).

^c^ Add, additive effect, which indicates the disease severity (%) decreased by QTL. The minus symbol “−” indicates that the susceptible parent ND495 contributed the resistance effect.

The 7D QTL *QFhb.rwg‐7D.2*, which was also contributed by ND495, was detected in both greenhouse experiments. This 7D QTL had the second largest additive effects (−9.21 in 18GH and −7.74 in 19GH), with LOD values of 7.11 (18GH) and 5.79 (19GH), and it explained 16% (18GH) and 14% (19GH) of the phenotypic variance. It peaked in a 6.3‐cM interval between *IWA8486* and *IWB14320* at position 64.3–71.7 cM (Figure 2; Table 4).

Contrary to the two QTL described above (*QFhb.rwg‐2D* and *QFhb.rwg‐7D.2*), the resistant allele at the other four QTL (*QFhb.rwg‐1D*, *QFhb.rwg‐5B*, *QFhb.rwg‐7D.1*, and *QFhb.rwg‐7D.3*) were all derived from the resistant parent Largo, but they were detected only in single environments. *QFhb.rwg‐1D, QFhb.rwg‐5B*, and *QFhb.rwg‐7D.3* were detected in the field environment in 2019 (19F) and explained 11%, 10%, and 8% of the phenotypic variation, respectively. *QFhb.rwg‐7D.1* was detected in the field environment in 2018 (18F) and explained 9% of the phenotypic variation. *QFhb.rwg‐1D* peaked in a 3.2‐cM interval between *IWB17549* and *IWB6869* at position 45.4–48.6 cM in the centromere region on chromosome 1D (Figure 2; Table 4). *QFhb.rwg‐5B*, located on the long arm of chromosome 5B, had the highest LOD value at marker *IWB52117* at position 77.8 cM (Figure 2; Table 4). The QTL *QFhb.rwg‐7D.1* peaked at marker *IWB15280* at the 42.2 cM position on the short arm of chromosome 7D, whereas *QFhb.rwg‐7D.3* was mapped at marker *IWB8024* at position 88.2 cM on the long arm of the same chromosome (Figure 2; Table 4).

The significant effects of *QFhb.rwg‐1D*, *QFhb.rwg‐2D*, and *QFhb.rwg‐7D.2* were confirmed using the QTLNetwork program. However, the markers at the other three QTL (*QFhb.rwg‐5B*, *QFhb.rwg‐7D.1*, and *QFhb.rwg‐7D.3*) that showed only minor association with FHB resistance using the program QGene v.4.4.0 did not reach the significant level when data were analyzed with QTLNetwork program. The results also showed that no epistatic interactions were detected among the three QTLs *QFhb.rwg‐1D*, *QFhb.rwg‐2D*, and *QFhb.rwg‐7D.2*.

**FIGURE 3:**
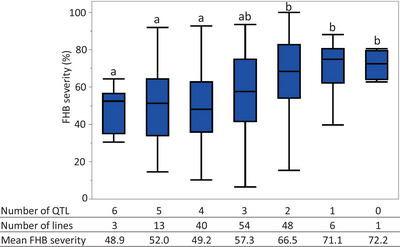
Box plot diagram of average Fusarium head blight (FHB) severities among the quantitative trait loci (QTL) groups in the ND495 × Largo population tested in two greenhouse seasons and two field experiments in 2018 and 2019. The means with the same letters on the top of the whiskers did not differ significantly (*α* < 0.05) as determined by least significant difference (LSD).

Among 188 RILs, 165 had no missing allele information at the significant markers, and they were grouped based on their QTL haplotypes. The QTL groups on the box plot diagrams were represented by markers with the highest LOD value: *IWB6869*, *IWB484*, *IWB52117*, *IWB15280*, *IWB14320*, and *IWB8024* for *QFhb.rwg‐1D*, *QFhb.rwg‐2D*, *QFhb.rwg‐5B*, *QFhb.rwg‐7D.1*, *QFhb.rwg‐7D.2*, and *QFhb.rwg‐7D.3*, respectively. Box plot graphs comparing QTL groups across environments (Figure S1 and Figure 3) reflected the distribution of FHB severity shown in the histograms in Figure 1. The differences among the QTL groups were smaller in the 18F and 19F data sets than those in the 18GH, 19GH, and overall average data set (Figure S1 and Figure 3). As expected, the groups carrying 4–6 QTL generally had lower FHB severities (average: 53.4%, range 43.0%–74.7%) than those having 0–3 QTL (average: 64.3%, range 51.4%–72.4%); the groups carrying the two main QTL, namely, *QFhb.rwg‐2D* and *QFhb.rwg‐7D.2* from ND495, had lower FHB severities (average: 49.8%, range 43.0%–54.7%) than those without the two QTL (average: 70.4%, range 61.7%–74.7%) (Figure S1 and Figure 3).

Although the QTL from Largo had less effect on FHB severity, they clearly played an important role in enhancing FHB resistance. For instance, among the groups carrying one main QTL from ND495, the groups carrying 2–4 QTL from Largo had lower FHB severities (range 49.7%–58.8%) than those with 0–1 Largo QTL (range 65.5%–71.1%) (Figure S1 and Figure 3). From this analysis, we also observed that the average disease severity of the QTL group carrying all six resistance alleles (48.9%) was slightly higher than the group carrying two resistance alleles from each parent (43.0%) (Figure S1 and Figure 3). Meanwhile, the group carrying all four QTL from Largo had the highest FHB severity (74.7%). These discrepancies are likely caused by the differences in the number of lines and/or genetic backgrounds.

The markers at the three minor QTL (*QFhb.rwg‐5B*, *QFhb.rwg‐7D.1*, and *QFhb.rwg‐7D*.3) aligned with the Chinese Spring reference genome v2.1 at genes *TraesCS5B03G1216000*, *TraesCS7D03G0166600*, and *TraesCS7D03G0942700*. The functions of these genes were described as unknown or likely non‐disease resistance related. The sequence of marker *IWB17549* at *QFhb.rwg‐1D* did not give significant alignment on chromosome 1D using the Chinese Spring reference genome v2.1. Although 845 genes were identified in the 76,868,328 bp–252,729,664 bp region using the flanking marker *IWB49565*, only seven of them encode nucleotide‐binding site leucine‐rich repeat (NBS‐LRR) proteins. Out of the seven genes, two (*TraesCS1D03G0448900* and *TraesCS1D03G0449300*) were within 1–2 Mb distance from the significant marker *IWB6859* (252,729,563–252,729,664 bp) (Table S8). A total of 476 genes were identified in the *QFhb.rwg‐2D* region between the significant markers *IWA4789* and *IWB18804* (483,825,998–541,262,766 bp), six of which have NBS‐LRR functions (Table S9). No gene was found with NBS‐LRR function out of the 226 genes identified in the *QFhb.rwg‐7D.2* region (Table S10). In addition to the high‐confidence genes mentioned above, 1644, 507, and 264 low‐confidence genes were identified in the *QFhb.rwg‐1D*, *QFhb.rwg‐2D*, and *QFhb.rwg‐7D.2* regions, respectively, including 7, 1, and 3 NBS‐LRR genes (Tables S8–S10).

### 3.4 Development and validation of KASP markers linked to identified QTL

Ten KASP markers were developed to track the six FHB resistance QTL identified in the ND495/Largo RIL population (Table 1). The KASP markers were validated in the ND495/Largo RIL population and in a set of 25 SHW lines with moderate and high levels of FHB resistance (Figure S2). The marker allele information of ND495/Largo RIL population and 25 SHW lines, along with their FHB severities from Szabo‐Hever et al. (2018), were shown in Tables S11 and S12, respectively. The results demonstrated that the marker alleles of the 10 KASP markers in the ND495/Largo RIL population were highly consistent with those of 9K or 90K SNP markers (Table S11). The similarity between 9K/90K SNPs and their respective KASP markers was 96.7% for three markers (*KASP‐IWB6869‐1D* and *KASP‐IWA4789‐2D*, and *KASP‐IWB52117‐5B*), 97.3% for four markers (*KASP‐IWB57869‐2D*, *KASP‐IWB15280‐7D*, *KASP‐IWB14320‐7D*, and *KASP‐IWB8024‐7D*), 97.8% for two markers (*KASP‐IWB17549‐1D*, *KASP‐IWA8486‐7D*), and 98.9% for one marker (*KASP‐IWB484‐2D*), suggesting that these KASP markers could be useful in marker‐assisted selection.

All the resistant SHW lines reported by Szabo‐Hever et al. (2018) have the resistance alleles of two KASP markers (*KASP‐IWB17549‐1D* and *KASP‐IWB6869‐1D*) for *QFhb.rwg‐1D* and one marker (*KASP‐IWB8024‐7D*) for *QFhb.rwg‐7D.3* (Table S12). Interestingly, only 40% of the SHW lines carried the resistance alleles of the markers (*KASP‐IWB52117‐5B* and *KASP‐IWB15280‐7D*) for *QFhb.rwg‐5B* and *QFhb.rwg‐7D.1*. Except for one SHW line, SW187 (pedigree: *T. dicoccum* PI 272527/*Ae*. *tauschii* CIae 26), all other SHW lines with *Ae*. *tauschii* Clae 26 in their pedigrees had the resistance allele of the marker *FHB‐IWB484‐2D* at *QFhb.rwg‐2D* region. All SHW lines with *Ae*. *tauschii* accessions RL 5286 and PI 268210 in their pedigrees had the resistance allele of the marker *FHB‐IWB15280‐7D.1* at *QFhb.rwg‐7D.1* region. Also, the two SHW lines SW92 and SW188, having *Ae*. *tauschii* RL 5286 in their pedigree, carried resistance allele of marker *FHB‐IWA8486‐7D* at *QFhb.rwg‐7D.2* region.

## 4 DISCUSSION

In modern wheat germplasm improvement, the development of SHW germplasm is a useful approach for introducing novel genes and alleles from *Ae. tauschii* and tetraploid wheat germplasm into common wheat. In this aspect, the development and utilization of SHW line Largo for breeding greenbug resistance represents one of the pioneering and successful breeding practices. Largo was developed in the late 1970s and released as germplasm carrying *Gb3* for resistance to greenbug biotype E (Joppa & Williams, 1982; Joppa et al., 1980). From 1980 to 1984, Largo was used as the source of *Gb3* for the development of the winter wheat variety TAM 110 (pedigree: ‘TAM 105′*4/Amigo*5//Largo) in Texas (Lazar et al., 1997). TAM 110 was further used as the source of *Gb3* for other most widely grown varieties in the state, such as TAM 112 (Rudd et al., 2014) and TAM 204 (Rudd et al., 2019). Recently, Largo's *Ae. tauschii* parent, PI 268210 (TA 1618), was reported to contribute the gene *Cmc_TAM112_* for resistance to wheat curl mite (*Aceria tosichella* Keifer), a vector for wheat streak mosaic virus (genus *Tritimovirus*) (Dhakal et al., 2018; Gaurav et al., 2022).

In addition to resistance to greenbug and curl mite, Largo was also found to have resistance to several other important wheat diseases (Adhikari et al., 2015; Friesen et al., 2008; Sharma et al., 2021; Szabo‐Hever et al., 2018). By using the ND495/Largo RIL population, Adhikari et al. (2015) detected four QTL for resistance to septoria tritici blotch, caused by *Zymoseptoria tritici* (Desm.) Quaedvlieg and Crous (syn: *Mycosphaerella graminicola* (Fuckel) Schrot). Sharma et al. (2021) identified six stem rust resistance genes, with two being potentially novel genes or alleles. In the present study, we identified four Largo‐derived FHB resistance QTL, further demonstrating that Largo is a valuable SHW germplasm for wheat improvement. Although a number of SHW lines were previously reported to have resistance to FHB (Das et al., 2016; Oliver et al., 2005; Szabo‐Hever et al., 2018; Zhu et al., 2016), molecular mapping of FHB resistance in SHW germplasm has not previously been reported. Therefore, this study may represent the first effort to dissect FHB resistance in synthetic wheat germplasm.

Among the six FHB resistance QTL identified in this study, *QFhb.rwg‐1D* was mapped to the centromeric region of chromosome 1D and derived from the resistant parent Largo. Based on its location, *QFhb.rwg‐1D* should be different from the QTL previously mapped to the distal end of chromosome arm 1DS in common wheat (Ittu et al., 2000; Klahr et al., 2007; Liu et al., 2013; Lu et al., 2013; Z. Yang et al., 2005). Islam et al. (2016) identified a QTL in the centromeric region of chromosome 1D from the winter wheat variety Truman that has excellent FHB resistance. Petersen et al. (2016) detected a QTL near the centromeric region of chromosome 1D from the susceptible wheat variety AGS 2000 in a RIL population. Although these QTL may coincide, *QFhb.rwg‐1D* from Largo and the 1D QTL from Truman and AGS 2000 are likely different. First, *QFhb.rwg‐1D* was identified from a resistant parent based on FHB disease severity, and although the 1D QTL from Truman was also identified from a resistant parent, it was only associated with reduced incidence (Islam et al., 2016). Second, the 1D QTL from AGS 2000 was identified from a susceptible parent, and it was only associated with decreased FDK and DON (Petersen et al., 2016).

The QTL *QFhb.rwg‐2D* originated from ND495, and it was mapped in the proximal region (close to the centromere) of chromosome arm 2DL. Another FHB resistance QTL (*QFhs.crc‐2DL*) in this chromosome region was first identified in the Chinese wheat variety Wuhan 1 (Somers et al., 2003). Thereafter, a major and stable FHB resistance QTL has been detected in this chromosomal region in many wheat varieties and lines, including Chinese wheat landrace Wangshuibai (Lin et al., 2006) and line CJ 9306 (Jiang, Dong, et al., 2007; Jiang, Shi, et al., 2007), Chinese wheat variety Ji5265 (H. Li et al., 2022), US soft red winter wheat breeding line VA00W‐38 (Liu et al., 2012), CIMMYT wheat line Shanghai‐3/Catbird (Lu et al., 2013), SHW derived lines Soru#1 (He et al., 2016) and C615 (Yi et al., 2018), and Canadian spring wheat varieties Kenyon (McCartney et al., 2016), AAC Tenacious (Dhariwal et al., 2020), AC Barrie (Thambugala et al., 2020), and the elite Chinese wheat cultivar Yangmai 158 (Yan et al., 2021). The three QTL identified based on SSR markers in Wuhan 1, CJ 9306, and Shanghai‐3/Catbird all linked to SSR marker *gwm539* (Jiang, Dong, et al., 2007; Jiang, Shi, et al., 2007; Lu et al., 2013; Somers et al., 2003). The 2DL QTL identified in Soru#1 (He et al., 2016) using 90K SNP and SSR markers was mapped within the marker interval *gwm539‐*‐*IWB44589* (*Kukri_c36639_186*), which corresponds to the physical interval of 513–574 Mb on chromosome arm 2DL in the Chinese Spring RefSeq v1.0 sequence (IWGSC, 2018; Zheng et al., 2021). F. Zhang et al. (2024) recently identified that the 2DL QTL derived from a Chinese wheat variety, Shi4185 is the same QTL as *QFhb‐2DL* in Ji5265 and designated the QTL as *Fhb9*. They mapped *Fhb9* to an 8.0 Mb (525.9–533.8 Mb) interval. Because *QFhb.rwg‐2D* peaked at the marker interval *IWA4789*–*IWB18804*, which corresponds to the physical interval of 481.6–538.7 Mb (IWGSC, 2018; Zheng et al., 2021), it physically overlapped or co‐localized with the *Fhb9* or 2DL QTL identified in the wheat varieties or lines described above. Therefore, *QFhb.rwg‐2D* is likely the same as *Fhb9*, present in many wheat varieties on chromosome 2D. However, because *QFhb.rwg‐2D* is derived from the susceptible parent and has not been fine mapped, we could not rule out the possibility that *QFhb.rwg‐2D* is a QTL closely linked to *Fhb9* in this region.

The *QFhb.rwg‐5B* from Largo is located on the long arm of chromosome 5B. Several FHB resistance QTL have been reported on chromosome arm 5BL (see reviewed by H. Buerstmayr et al., 2009; Liu et al., 2009; Petersen et al., 2016; R. Wang et al., 2017; Zheng et al., 2021). Based on 30 original QTL in previous studies and meta‐QTL analysis, Zheng et al. (2021) identified six sequence‐based high‐confidence mQTL (smQTL), named *sMQTL‐5B‐2* through *sMQTL‐5B‐7*, on chromosome arm 5BL. *QFhb.rwg‐5B* is physically located within the interval of 666.2–669.6 Mb between markers *IWB71749* and *IWB62048* on chromosome 5B in the Chinese Spring RefSeq v1.0.sequence (IWGSC, 2018; Zheng et al., 2021), and it is positioned between *sMQTL‐5B‐6* (610–623 Mb) and *sMQTL‐5B‐7* (679–680 Mb) (Zheng et al., 2021). Since *QFhb.rwg‐5B* does not physically overlap with any of the six smQTL, it is likely a different QTL. However, because of the close proximity, we cannot rule out the possibility that *QFhb.rwg‐5B* is the same QTL as *sMQTL‐5B‐*7 or any of its five original QTL identified in the wheat variety Patterson (Bourdoncle & Ohm, 2003), Georgian spelt wheat (*T*. *macha*), and the Canadian spring wheat variety Kenyon (McCartney et al., 2016). In addition, Ghavami et al. (2011) identified a QTL (*Qfhs.ndsu‐5BL*) on chromosome arm 5BL in durum wheat. Given that *QFhb.rwg‐5B* in Largo also originated from durum (Langdon), it was theorized that *QFhb.rwg‐5B* may be the same QTL as *Qfhs.ndsu‐5BL*, but comparing the positions of the two QTL in the Svevo reference genome v1.0 (Maccaferri et al., 2019), *Qfhs.ndsu‐5BL* is likely about 20–35 Mb more distal from *QFhb.rwg‐5B*.

Three FHB resistance QTL (*QFhb.rwg‐7D.1*, *QFhb.rwg‐7D.2*, and *QFhb.rwg‐7D.3*) were identified on chromosome 7D, with one having a large effect (*QFhb.rwg‐7D.2*) and two having minor effects (*QFhb.rwg‐7D.1* and *QFhb.rwg‐7D.3*) derived from ND495 and Largo, respectively. Several FHB resistance QTL were previously mapped onto chromosome 7D in different bread wheat materials, including recently designated *Fhb8* (*Qfdk.nau‐7D*) for reducing Fusarium‐damaged kernels (FDK) identified in Wangshuibai (X. Wang et al., 2024). By using meta‐QTL and sequence analysis, Zheng et al. (2021) consolidated 17 QTL from chromosome 7D into four smQTL: *sMQTL‐7D‐1* (90–102 Mb), *sMQTL‐7D‐2* (124–144 Mb), *sMQTL‐7D‐3* (168–194 Mb), and *sMQTL‐7D‐4* (506–518 Mb), which consist of two, five, three, and seven original QTL, respectively. In the Chinese Spring reference genome v1.0, the two significant markers *IWA8486* (117.8 Mb) and *IWB14320* (146.1 Mb) for *QFhb.rwg‐7D.2* span an interval covering the *sMQTL‐7D‐2* (124–144 Mb) region, indicating that *QFhb.rwg‐7D.2* is likely the same QTL as those mapped to the *sMQTL‐7D‐2* region close to the centromeric region of chromosome arm 7DS in Wangshuibai (Jia et al., 2005; C. Li et al., 2008), Riband (Draeger et al., 2007), Haiyanzhong (Cai et al., 2016; T. Li et al., 2011), Catbird (Cativelli et al., 2013), Kenyon (McCartney et al., 2016), and several breeding lines (Eckard et al., 2015).

The *QFhb.rwg‐7D.1* on 7DS is situated about 20 cM distal to *QFhb.rwg‐7D.2*. The significant marker *IWB15280* for *QFhb.rwg‐7D.1* was physically located at 42.5 Mb and flanked by markers *IWB18267* (40.3 Mb) and *IWB16984* (43.0 Mb) (Chinese Spring reference genome v1.0). This region has not been previously shown to be associated with FHB resistance, except for the two QTL that were previously mapped about 15 cM proximal and distal to *QFhb.rwg‐7D.1* (T. Li et al., 2011; Ren et al., 2019; Szabo‐Hever et al., 2014), suggesting that *QFhb.rwg‐7D.1* may be a novel QTL. The significant marker *IWB8024* (516.0 Mb) for *QFhb.rwg‐7D.3* on 7DL was flanked by *IWB16157* and *IWB16883* in an interval of 503.7–526.4 Mb (Chinese Spring reference genome v1.0), which coincides with the *sMQTL‐7D‐4* (506–518 Mb) region, indicating that *QFhb.rwg‐7D.3* might be the same QTL as those mapped to the *sMQTL‐7D‐4* region. In addition, Buerstmayr and Buerstmayr (2015) mapped an FHB resistance QTL in Capo on the long arm of chromosome 7D that was in close proximity to *QFhb.rwg‐7D.3*. These two QTL cannot be directly compared due to the absence lack of common markers between the two maps, but indirect comparison by previously published map data indicates that they are likely the same (Blake et al., 2019; Ren et al., 2019).

Among the six QTL, four (*QFhb.rwg‐1D*, *QFhb.rwg‐5B*, *QFhb.rwg‐7D.1*, and *QFhb.rwg‐7D.3*) were derived from the resistant parent Largo, and two (*QFhb.rwg‐2D* and *QFhb.rwg‐7D.2*) were derived from the susceptible parent ND495. In bi‐parent populations, FHB resistance QTL are usually identified from the resistant parents. However, they are also frequently identified from susceptible parents; for example, resistance QTL have been reported on chromosome 2A in the susceptible durum varieties Ben (Q. Zhang et al., 2014) and Joppa (Zhao et al., 2018), on chromosome 1B in the winter wheat variety Lynx (Schmolke et al., 2005), and on chromosome 3A in the spring wheat variety AC Foremost. Susceptibility in wheat genotypes carrying FHB‐resistance QTL can be caused by suppressors or epistatic susceptibility genes. G. Li et al. (2023) recently identified two suppressors, *In1* and *In2*, which inhibit *Fhb1*‐mediated resistance to FHB. In a survey of 1601 wheat accessions, G. Li et al. (2023) found that *In1* and *In2* are commonly present in over 50% of the accessions worldwide. Specifically, G. Li et al. (2023) reported that 66% of 100 varieties and lines from North and South America, including 42 from the United States, carry at least one of these suppressors. Given their widespread distribution, *In1* and/or *In2* could possibly be present in ND495; however, this remains to be investigated. Moreover, it is currently unclear whether *In1* and *In2* can suppress FHB resistance conferred by genes or QTL other than *Fhb1*.

Several studies have shown that the susceptible factors are commonly present in both tetraploid and hexaploid wheat on chromosome arms 7AS, 3DL, and 4DS (Chhabra et al., 2021; Fabre et al., 2020; Garvin et al., 2015; Hales et al., 2020; Ma et al., 2006). Garvin et al. (2015) and Hales et al. (2020) demonstrated that deletion of the factors on chromosome arms 3DL and 7AS increased FHB resistance. Su et al. (2019) demonstrated that a histidine‐rich calcium‐binding protein gene (*TaHRC*), which was identified as the causal gene for *Fhb1*, is an FHB‐susceptible factor. The *Fhb1*‐mediated FHB resistance extensively used in wheat breeding and production in fact resulted from natural mutation caused by a large deletion in the second intron and the beginning of the third exon of the *TaHRC* susceptible allele. Su et al. (2019) and Chen, Su, Tian, Hao et al. (2022), Chen, Su, Tian, Liu, et al. (2022) showed that knockout of the *TaHRC* susceptible allele in the FHB‐susceptible variety Bobwhite through gene editing improved FHB resistance. In the present study, the two QTL (*QFhb.rwg‐2D* and *QFhb.rwg‐7D.2*) from the susceptible parent ND495 had larger resistance effects than the four QTL from the resistance parent Largo, suggesting that ND495 might possess suppressor(s) or susceptibility gene(s) that may suppress or mask resistance controlled by the resistance QTL. In the bi‐parental segregating population, the FHB resistance QTL derived from the susceptible parent ND495 may have been separated from their suppressor(s) or susceptibility factors in a subset of genotypes due to independent assortment and recombination, thereby enabling the expression of the resistance QTL contributed by the susceptible parent. In our study, no significant epistatic effect was discovered between the QTL identified in Largo and ND495. Therefore, further research may be necessary to discover additional QTL or genes associated with FHB resistance or susceptibility.

FHB resistance in wheat has been shown to share pleiotropic loci with several morphological traits, including PH, heading or FD, spike morphology, and anther retention/extrusion (Akohoue et al., 2022; Buerstmayr et al., 2020). Consequently, it is possible for QTL mapping studies targeting FHB resistance to identify loci associated with traits other than FHB resistance per se. In the present study, we posit that the QTL on chromosomes 2D and 7D, identified from the susceptible parent ND495, are unlikely to be associated with PH or FD, given the absence or very low correlations between FHB severity and these traits in the two field experiments. However, the potential association of these QTL with spike morphology or anther retention/extrusion remains unclear, as these traits were not assessed in this study.

It is notable that all four minor QTL identified from Largo were detected exclusively in a single field environment and were not observed in the greenhouse experiments. Unlike greenhouse assays, which primarily evaluate Type II resistance (resistance to fungal spread within the spike), field experiments can capture both Type I resistance (resistance to initial infection) and Type II resistance. Therefore, it is possible that some of the minor QTL detected only under field conditions are specifically associated with Type I resistance. The lack of repeatability of these QTL across the two field environments may be attributed to differing weather conditions, as we observed that disease pressure was considerably higher in 2019 compared to 2018 (Figure 1; Table 2), likely due to excessive rainfall in 2019. Given the inherent complexity and variability of field experiments, these minor QTL require further validation in future studies.

In summary, Largo (Langdon × *Ae*. *tauschii* PI 268210) was identified to possess a moderate to high level of FHB resistance in our previous experiments (Szabo‐Hever et al., 2018). In the current study, we detected three (*QFhb.rwg‐1D*, *QFhb.rwg‐7D.1*, and *QFhb.rwg‐7D.3*) and one (*QFhb.rwg‐5B*) QTL originating from *Ae*. *tauschii* PI 268210 and Langdon, respectively, with *QFhb.rwg‐1D* and *QFhb.rwg‐7D.1* likely being novel QTL. Therefore, FHB resistance in Largo is confirmed to result from the mutual action of the four minor QTL. An unexpected discovery was the identification of two QTL with larger effects on chromosomes 2D (*QFhb.rwg‐2D*) and 7D (*QFhb.rwg‐7D.2*) from ND495. Furthermore, *QFhb.rwg‐2D*, which had the largest effect, is believed to be the major FHB resistance 2D QTL or *Fhb9* found in various wheat varieties and lines. Since no major FHB resistance QTL was previously detected on chromosome 2D in hard red spring wheat germplasm adapted to the spring wheat region in the U.S., the identification of *QFhb.rwg‐2D* in ND495 will facilitate its integration into breeding programs for hard red spring wheat. Moreover, identifying these QTL in both the resistant and susceptible parents suggests that the optimal strategy for enhancing FHB resistance is to combine multiple resistance QTL while simultaneously eliminating susceptibility genes using the KASP markers developed and validated in this study.

## AUTHOR CONTRIBUTIONS

**Agnes Szabo‐Hever**: Data curation; formal analysis; investigation; methodology; validation; writing—original draft; writing—review and editing. **Jyoti Sharma**: Data curation; formal analysis; investigation; methodology; writing—review and editing. **Justin D. Faris**: Data curation; formal analysis; investigation; methodology; supervision; visualization; writing—review and editing. **Shaobin Zhong**: Data curation; formal analysis; funding acquisition; investigation; methodology; resources; validation; visualization; writing—review and editing. **Timothy L. Friesen**: Data curation; investigation; methodology; resources; writing—review and editing. **Jason D. Fiedler**: Data curation; investigation; methodology; resources; validation; writing—review and editing. **Andrew J. Green**: Data curation; investigation; methodology; resources; visualization; writing—review and editing. **Guihua Bai**: Data curation; formal analysis; investigation; methodology; validation; writing—review and editing. **Elias M. Elias**: Data curation; formal analysis; investigation; methodology; resources; supervision; writing—review and editing. **Steven S. Xu**: Conceptualization; data curation; formal analysis; funding acquisition; investigation; methodology; project administration; resources; software; supervision; validation; visualization; writing—original draft; writing—review and editing.

## CONFLICT OF INTEREST STATEMENT

The authors declare no conflicts of interest.

## Supporting information




**Supplemental Table S1**. Whole genome linkage map for the ND495 × Largo recombinant inbred line population.
**Supplemental Table S2**. Genotypic data for ND495 × Largo population of 188 recombinant inbred lines genotyped with the wheat Infinium 90K SNP iSelect array and SSR markers.
**Supplemental Table S3**. Least significant difference (LSD) tests for the Fusarium head blight (FHB) severity data of the ND495 × Largo recombinant inbred lines, their parents, and the resistant and susceptible checks (Sumai 3 and Wheaton, respectively) from the greenhouse experiment in 2018 (18GH).
**Supplemental Table S4**. Least significant difference (LSD) tests for the Fusarium head blight (FHB) severity data of the ND495 × Largo recombinant inbred lines, their parents, and the resistant and susceptible checks (Sumai 3 and Wheaton, respectively) from the greenhouse experiment in 2019 (19GH).
**Supplemental Table S5**. Least significant difference (LSD) tests for the Fusarium head blight (FHB) severity data of the ND495 × Largo recombinant inbred lines, their parents, and the resistant and susceptible checks (Sumai 3 and Wheaton, respectively) from the field experiment in 2018 (18F).
**Supplemental Table S6**. Least significant difference (LSD) tests for the Fusarium head blight (FHB) severity data of the ND495 × Largo recombinant inbred lines, their parents, and the resistant and susceptible checks (Sumai 3 and Wheaton, respectively) from the field experiment in 2019 (19F).
**Supplemental Table S7**. Number of markers and length for each chromosome of the ND495/Largo RIL population.
**Supplemental Table S8**. List of candidate genes in the *QFhb.rwg‐1D* region on chromosome 1D.
**Supplemental Table S9**. List of candidate genes in the *QFhb.rwg‐2D* region on chromosome 2D.
**Supplemental Table S10**. List of candidate genes in the *QFhb.rwg‐7D.2* region on chromosome 7D.
**Supplemental Table S11**. Comparison of allele information derived from significant SNP markers and their corresponding KASP markers.
**Supplemental Table S12**. Allele information of KASP markers on the 25 resistant SHW lines published by Szabo‐Hever et al. (2018). For comparison, the resistant allele, susceptible allele, ND495, and Largo genotype information from the current study are listed.
**Supplemental Figure S1**. Box plot diagrams of FHB severities (%) of the QTL groups of the ND495/Largo RILs in each of the environments. 18GH, 19GH, 18F, and 19F are FHB severity data from greenhouse and field experiments in 2018 and 2019, respectively. FHBALL are average FHB severity data from 18F, 19F, 18GH, and 19GH experiments. The means with the same letters on the top of the whiskers did not differ significantly (α < 0.05) as determined by LSD.
**Supplemental Figure S2**. Validation of KASP markers linked to FHB resistance QTL *QFhb.rwg‐1D*, *QFhb.rwg‐2D*, and *QFhb.rwg‐5B* in the ND495/Largo RIL population and the 25 resistant SHW lines published by Szabo‐Hever et al. (2018). The Letters “R” and “S” represent resistant and susceptible alleles, respectively.

## Data Availability

All data and datasets generated or analyzed during this study are included in this published article and its Supporting Information. Referenced data are available in the literature.

## References

[tpg270073-bib-0001] Adhikari, T. B. , Mamidi, S. , Gurung, S. , & Bonman, J. M. (2015). Mapping of new quantitative trait loci (QTL) for resistance to Septoria tritici blotch in spring wheat (*Triticum aestivum* L.). Euphytica, 205, 699–706. 10.1007/s10681-015-1393-4

[tpg270073-bib-0002] Ágnes, S.‐H. , Szabolcs, L.‐K. , Mónika, V. , László, P. , János, P. , Csaba, L. , & Ákos, M. (2014). Differential influence of QTL linked to Fusarium head blight, Fusarium‐damaged kernel, deoxynivalenol contents and associated morphological traits in a Frontana‐derived wheat population. Euphytica, 200, 9–26. 10.1007/s10681-014-1124-2

[tpg270073-bib-0003] Akohoue, F. , Koch, S. , Plieske, J. , & Miedaner, T. (2022). Separation of the effects of two reduced height (*Rht*) genes and genomic background to select for less Fusarium head blight of short‐strawed winter wheat (*Triticum aestivum* L.) varieties. Theoretical and Applied Genetics, 135, 4303–4326. 10.1007/s00122-022-04219-4 36152062 PMC9734223

[tpg270073-bib-0004] Andersen, A. L. (1948). The development of *Gibberella zeae* head blight of wheat. Phytopathology, 38, 595–611.

[tpg270073-bib-0005] Blake, V. C. , Woodhouse, M. R. , Lazo, G. R. , Odell, S. G. , Wight, C. P. , Tinker, N. A. , Wang, Y. , Gu, Y. Q. , Birkett, C. L. , Jannink, J.‐L. , Matthews, D. E. , Hane, D. L. , Michel, S. L. , Yao, E. , & Sen, T. Z. (2019). GrainGenes: Centralized small grain resources and digital platform for geneticists and breeders. Database, 2019. 10.1093/database/baz065 PMC6580076 31210272

[tpg270073-bib-0006] Bottalico, A. , & Perrone, G. (2002). Toxigenic *Fusarium* species and mycotoxins associated with head blight in small‐grain cereals in Europe. In Mycotoxins in plant disease (pp. 611–624). Springer. 10.1007/978-94-010-0001-7_2

[tpg270073-bib-0007] Bourdoncle, W. , & Ohm, H. W. (2003). Quantitative trait loci for resistance to Fusarium head blight in recombinant inbred wheat lines from the cross Huapei 57‐2/Patterson. Euphytica, 131, 131–136. 10.1023/A:1023056207513

[tpg270073-bib-0008] Brisco, E. I. , Brown, L. K. , & Olson, E. L. (2017). Fusarium head blight resistance in *Aegilops tauschii* . Genetic Resources and Crop Evolution, 64, 2049–2058. 10.1007/s10722-017-0495-3

[tpg270073-bib-0009] Buerstmayr, H. , Ban, T. , & Anderson, J. A. (2009). QTL mapping and marker‐assisted selection for Fusarium head blight resistance in wheat: A review. Plant Breeding, 128, 1–26. 10.1111/j.1439-0523.2008.01550.x

[tpg270073-bib-0010] Buerstmayr, H. , Stierschneider, M. , Steiner, B. , Lemmens, M. , Griesser, M. , Nevo, E. , & Fahima, T. (2003). Variation for resistance to head blight caused by *Fusarium graminearum* in wild emmer (*Triticum dicoccoides*) originating from Israel. Euphytica, 130, 17–23. 10.1023/A:1022324727780

[tpg270073-bib-0011] Buerstmayr, M. , Alimari, A. , Steiner, B. , & Buerstmayr, H. (2013). Genetic mapping of QTL for resistance to Fusarium head blight spread (type 2 resistance) in a *Triticum dicoccoides* × *Triticum durum* backcross‐derived population. Theoretical and Applied Genetics, 126, 2825–2834. 10.1007/s00122-013-2174-x 23921957

[tpg270073-bib-0012] Buerstmayr, M. , & Buerstmayr, H. (2015). Comparative mapping of quantitative trait loci for Fusarium head blight resistance and anther retention in the winter wheat population Capo × Arina. Theoretical and Applied Genetics, 128, 1519–1530. 10.1007/s00122-015-2527-8 25982129 PMC4477076

[tpg270073-bib-0013] Buerstmayr, M. , Huber, K. , Heckmann, J. , Steiner, B. , Nelson, J. C. , & Buerstmayr, H. (2012). Mapping of QTL for Fusarium head blight resistance and morphological and developmental traits in three backcross populations derived from *Triticum dicoccum* × *Triticum durum* . Theoretical and Applied Genetics, 125, 1751–1765. 10.1007/s00122-012-1951-2 22926291 PMC3493669

[tpg270073-bib-0014] Buerstmayr, M. , Steiner, B. , & Buerstmayr, H. (2020). Breeding for Fusarium head blight resistance in wheat—Progress and challenges. Plant Breeding, 139, 429–454. 10.1111/pbr.12797

[tpg270073-bib-0015] Cai, J. , Wang, S. , Li, T. , Zhang, G. , & Bai, G. (2016). Multiple minor QTLs are responsible for Fusarium head blight resistance in Chinese wheat landrace Haiyanzhong. PLoS One, 11, e0163292. 10.1371/journal.pone.0163292 27676181 PMC5038969

[tpg270073-bib-0016] Cativelli, M. , Lewis, S. , & Appendino, M. L. (2013). A Fusarium head blight resistance quantitative trait locus on chromosome 7D of the spring wheat cultivar Catbird. Crop Science, 53, 1464–1471. 10.2135/cropsci2012.07.0435

[tpg270073-bib-0017] Chen, H. , Su, Z. , Tian, B. , Hao, G. , Trick, H. N. , & Bai, G. (2022). *TaHRC* suppresses the calcium‐mediated immune response and triggers wheat Fusarium head blight susceptibility. Plant Physiology, 190, 1566–1569. 10.1093/plphys/kiac352 35900181 PMC9614457

[tpg270073-bib-0018] Chen, H. , Su, Z. , Tian, B. , Liu, Y. , Pang, Y. H. , Kavetskyi, V. , Trick, H. N. , & Bai, G. (2022). Development and optimization of a *Barley stripe mosaic virus* (BSMV)‐mediated gene editing system to improve Fusarium head blight (FHB) resistance in wheat. Plant Biotechnology Journal, 20, 1018–1020. 10.1111/pbi.13819 35348278 PMC9129070

[tpg270073-bib-0019] Chhabra, B. , Tiwari, V. , Gill, B. S. , Dong, Y. , & Rawat, N. (2021). Discovery of a susceptibility factor for Fusarium head blight on chromosome 7A of wheat. Theoretical and Applied Genetics, 134, 2273–2289. 10.1007/s00122-021-03825-y 33834252

[tpg270073-bib-0020] Chin, T. , Pleskach, K. , Tittlemier, S. A. , Henriquez, M. A. , Bamforth, J. , Gamage, N. W. , Ashfaq, T. , Lee, S.‐J. , Kurera, M. S. , Patel, B. , & Walkowiak, S. (2023). A status update on fusarium head blight on Western Canadian wheat. Canadian Journal of Plant Pathology, 45, 1–13. 10.1080/07060661.2023.2177352

[tpg270073-bib-0021] Chu, C. , Niu, Z. , Zhong, S. , Chao, S. , Friesen, T. L. , Halley, S. , Elias, E. M. , Dong, Y. , Faris, J. D. , & Xu, S. S. (2011). Identification and molecular mapping of two QTLs with major effects for resistance to Fusarium head blight in wheat. Theoretical and Applied Genetics, 123, 1107–1119. 10.1007/s00122-011-1652-2 21833554

[tpg270073-bib-0022] Cox, T. S. (1997). Deepening the wheat gene pool. Journal of Crop Production, 1, 1–25. 10.1300/J144v01n01_01

[tpg270073-bib-0023] Das, M. K. , Bai, G. , Mujeeb‐Kazi, A. , & Rajaram, S. (2016). Genetic diversity among synthetic hexaploid wheat accessions (*Triticum aestivum*) with resistance to several fungal diseases. Genetic Resources and Crop Evolution, 63, 1285–1296. 10.1007/s10722-015-0312-9

[tpg270073-bib-0024] Dhakal, S. , Tan, C. T. , Anderson, V. , Yu, H. , Fuentealba, M. P. , Rudd, J. C. , Haley, S. D. , Xue, O. , Ibrahim, A. M. H. , Garza, L. , Devkota, R. N. , & Liu, S. (2018). Mapping and KASP marker development for wheat curl mite resistance in “TAM 112” wheat using linkage and association analysis. Molecular Breeding, 38, 1–13. 10.1007/s11032-018-0879-x

[tpg270073-bib-0025] Dhariwal, R. , Henriquez, M. A. , Hiebert, C. , McCartney, C. A. , & Randhawa, H. S. (2020). Mapping of major *Fusarium* head blight resistance from Canadian Wheat cv. AAC Tenacious. International Journal of Molecular Sciences, 21, 4497. 10.3390/ijms21124497 32599868 PMC7350018

[tpg270073-bib-0026] Draeger, R. , Gosman, N. , Steed, A. , Chandler, E. , Thomsett, M. , Schondelmaier, J. , Buerstmayr, H. , Lemmens, M. , Schmolke, M. , Mesterhazy, A. , & Nicholson, P. (2007). Identification of QTLs for resistance to Fusarium head blight, DON accumulation and associated traits in the winter wheat variety Arina. Theoretical and Applied Genetics, 115, 617–625. 10.1007/s00122-007-0592-3 17607557

[tpg270073-bib-0027] Eckard, J. T. , Gonzalez‐Hernandez, J. L. , Caffe, M. , Berzonsky, W. , Bockus, W. W. , Marais, G. F. , & Baenziger, P. S. (2015). Native Fusarium head blight resistance from winter wheat cultivars ‘Lyman,’ ‘Overland,’ ‘Ernie,’ and ‘Freedom’ mapped and pyramided onto ‘Wesley’‐*Fhb1* backgrounds. Molecular Breeding, 35, 1–16. 10.1007/s11032-015-0200-1

[tpg270073-bib-0028] Fabre, F. , Rocher, F. , Alouane, T. , Langin, T. , & Bonhomme, L. (2020). Searching for FHB resistances in bread wheat: Susceptibility at the crossroad. Frontiers in Plant Science, 11, 731. 10.3389/fpls.2020.00731 32595664 PMC7300258

[tpg270073-bib-0029] Friesen, T. L. , Xu, S. S. , & Harris, M. O. (2008). Stem rust, tan spot, Stagonospora nodorum blotch, and Hessian fly resistance in Langdon durum–*Aegilops tauschii* synthetic hexaploid wheat lines. Crop Science, 48, 1062–1070. 10.2135/cropsci2007.08.0463

[tpg270073-bib-0030] Garvin, D. F. , Porter, H. , Blankenheim, Z. J. , Chao, S. , & Dill‐Macky, R. (2015). A spontaneous segmental deletion from chromosome arm 3DL enhances Fusarium head blight resistance in wheat. Genome, 58, 479–488. 10.1139/gen-2015-0088 26524120

[tpg270073-bib-0031] Gaurav, K. , Arora, S. , Silva, P. , Sánchez‐Martín, J. , Horsnell, R. , Gao, L. , Brar, G. S. , Widrig, V. , Raupp, W. J. , Singh, N. , Wu, S. , Kale, S. M. , Chinoy, C. , Nicholson, P. , Quiroz‐Chávez, J. , Simmonds, J. , Hayta, S. , Smedley, M. A. , Harwood, W. , … Wulff, B. B. H. (2022). Population genomic analysis of *Aegilops tauschii* identifies targets for bread wheat improvement. Nature Biotechnology, 40, 422–431. 10.1038/s41587-021-01058-4 PMC8926922 34725503

[tpg270073-bib-0032] Ghavami, F. , Elias, E. M. , Mamidi, S. , Ansari, O. , Sargolzaei, M. , Adhikari, T. , Mergoum, M. , & Kianian, S. F. (2011). Mixed model association mapping for Fusarium head blight resistance in Tunisian‐derived durum wheat populations. G3: Genes| Genomes| Genetics, 1, 209–218. 10.1534/g3.111.000489 22384332 PMC3276138

[tpg270073-bib-0033] Gladysz, C. , Lemmens, M. , Steiner, B. , & Buerstmayr, H. (2007). Evaluation and genetic mapping of resistance to Fusarium head blight in *Triticum dicoccoides* . Israel Journal of Plant Sciences, 55, 263–266. 10.1560/IJPS.55.3-4.263

[tpg270073-bib-0034] Haile, J. K. , N'Diaye, A. , Walkowiak, S. , Nilsen, K. T. , Clarke, J. M. , Kutcher, H. R. , Steiner, B. , Buerstmayr, H. , & Pozniak, C. J. (2019). Fusarium head blight in durum wheat: Recent status, breeding directions, and future research prospects. Phytopathology, 109, 1664–1675. 10.1094/PHYTO-03-19-0095-RVW 31369363

[tpg270073-bib-0035] Hales, B. , Steed, A. , Giovannelli, V. , Burt, C. , Lemmens, M. , Molnár‐Láng, M. , & Nicholson, P. (2020). Type II Fusarium head blight susceptibility conferred by a region on wheat chromosome 4D. Journal of Experimental Botany, 71, 4703–4714. 10.1093/jxb/eraa226 32473016 PMC7410183

[tpg270073-bib-0036] Hao, Y. , Rasheed, A. , Zhu, Z. , Wulff, B. B. , & He, Z. (2020). Harnessing wheat *Fhb1* for *Fusarium* resistance. Trends in Plant Science, 25, 1–3. 10.1016/j.tplants.2019.10.006 31679993

[tpg270073-bib-0037] He, X. , Lillemo, M. , Shi, J. , Wu, J. , Bjørnstad, Å. , Belova, T. , Dreisigacker, S. , Duveiller, E. , & Singh, P. (2016). QTL characterization of Fusarium head blight resistance in CIMMYT bread wheat line Soru# 1. PLoS One, 11, e0158052. 10.1371/journal.pone.0158052 27351632 PMC4924825

[tpg270073-bib-0038] Heffelfinger, C. , Fragoso, C. A. , & Lorieux, M. (2017). Constructing linkage maps in the genomics era with MapDisto 2.0. Bioinformatics, 33, 2224–2225. 10.1093/bioinformatics/btx177 28369214 PMC5870660

[tpg270073-bib-0039] IWGSC . (2018). Shifting the limits in wheat research and breeding using a fully annotated reference genome. Science, 361, eaar7191. 10.1126/science.aar7191 30115783

[tpg270073-bib-0040] Islam, M. , Brown‐Guedira, G. , Van Sanford, D. , Ohm, H. , Dong, Y. , & McKendry, A. L. (2016). Novel QTL associated with the Fusarium head blight resistance in Truman soft red winter wheat. Euphytica, 207, 571–592. 10.1007/s10681-015-1550-9

[tpg270073-bib-0041] Ittu, M. , Sãulescu, N. N. , Hagima, I. , Ittu, G. , & Mustãtea, P. (2000). Association of Fusarium head blight resistance with gliadin loci in a winter wheat cross. Crop Science, 40, 62–67. 10.2135/cropsci2000.40162x

[tpg270073-bib-0042] Jia, G. , Chen, P. , Qin, G. , Bai, G. , Wang, X. , Wang, S. , Zhou, B. , Zhang, S. , & Liu, D. (2005). QTLs for Fusarium head blight response in a wheat DH population of Wangshuibai/Alondra‘s’. Euphytica, 146, 183–191. 10.1007/s10681-005-9001-7

[tpg270073-bib-0043] Jiang, G. L. , Dong, Y. , Shi, J. , & Ward, R. W. (2007). QTL analysis of resistance to Fusarium head blight in the novel wheat germplasm CJ 9306. II. Resistance to deoxynivalenol accumulation and grain yield loss. Theoretical and Applied Genetics, 115, 1043–1052. 10.1007/s00122-007-0630-1 17726598

[tpg270073-bib-0044] Jiang, G. L. , Shi, J. , & Ward, R. W. (2007). QTL analysis of resistance to Fusarium head blight in the novel wheat germplasm CJ 9306. I. Resistance to fungal spread. Theoretical and Applied Genetics, 116, 3–13. 10.1007/s00122-007-0641-y 17898987

[tpg270073-bib-0045] Joehanes, R. , & Nelson, J. C. (2008). QGene 4.0, an extensible Java QTL‐analysis platform. Bioinformatics, 24, 2788–2789. 10.1093/bioinformatics/btn523 18940826

[tpg270073-bib-0046] Joppa, L. R. , Timian, R. G. , & Williams, N. D. (1980). Inheritance of resistance to greenbug toxicity in an amphiploid of *Triticum turgidum*/*T. tauschii* . Crop Science, 20, 343–344. 10.2135/cropsci1980.0011183x002000030013x

[tpg270073-bib-0047] Joppa, L. R. , & Williams, N. D. (1982). Registration of Largo, a greenbug resistant hexaploid wheat (Reg. No. GP 176). Crop Science, 22, 901–902. 10.2135/cropsci1982.0011183x002200040052x

[tpg270073-bib-0048] Klahr, A. , Zimmermann, G. , Wenzel, G. , & Mohler, V. (2007). Effects of environment, disease progress, plant height and heading date on the detection of QTLs for resistance to Fusarium head blight in an European winter wheat cross. Euphytica, 154, 17–28. 10.1007/s10681-006-9264-7

[tpg270073-bib-0049] Kosambi, D. D. (1943). The estimation of map distance from recombination values. Annuals of Eugenics, 12, 172–175. 10.1111/j.1469-1809.1943.tb02321.x

[tpg270073-bib-0050] Kumar, S. Stack, R. W. , Friesen, T. L. , & Faris, J. D. (2007). Identification of a novel Fusarium head blight resistance quantitative trait locus on chromosome 7A in tetraploid wheat. Phytopathology, 97, 592–597. 10.1094/PHYTO-97-5-0592 18943578

[tpg270073-bib-0051] Lazar, M. D. , Worrall, W. D. , Peterson, G. L. , Porter, K. B. , Rooney, L. W. , Tuleen, N. A. , Marshall, D. S. , McDaniel, M. E. , & Nelson, L. R. (1997). Registration of ‘TAM 110’ wheat. Crop Science, 37, 1978–1979. 10.2135/cropsci1997.0011183x003700060055x

[tpg270073-bib-0052] Lev‐Yadun, S. , Gopher, A. , & Abbo, S. (2000). The cradle of agriculture. Science, 288, 1602–1603. 10.1126/science.288.5471.1602 10858140

[tpg270073-bib-0053] Li, C. , Zhu, H. , Zhang, C. , Lin, F. , Xue, S. , Cao, Y. , Zhang, Z. , Zhang, L. , & Ma, Z. (2008). Mapping QTLs associated with Fusarium‐damaged kernels in the Nanda 2419 × Wangshuibai population. Euphytica, 163, 185–191. 10.1007/s10681-007-9626-9

[tpg270073-bib-0054] Li, G. , Yuan, Y. , Zhou, J. , Cheng, R. , Chen, R. , Luo, X. , Shi, J. , Wang, H. , Xu, B. , Duan, Y. , Zhong, J. , Wang, X. , Kong, Z. , Jia, H. , & Ma, Z. (2023). FHB resistance conferred by *Fhb1* is under inhibitory regulation of two genetic loci in wheat (*Triticum aestivum* L.). Theoretical and Applied Genetics, 136, 134. 10.1007/s00122-023-04380-4 37217699

[tpg270073-bib-0055] Li, H. , Zhang, F. , Zhao, J. , Bai, G. , Amand, P. St. , Bernardo, A. , Ni, Z. , Sun, Q. , & Su, Z. (2022). Identification of a novel major QTL from Chinese wheat cultivar Ji5265 for *Fusarium* head blight resistance in greenhouse. Theoretical and Applied Genetics, 135, 1867–1877. 10.1007/s00122-022-04080-5 35357527

[tpg270073-bib-0056] Li, T. , Bai, G. , Wu, S. , & Gu, S. (2011). Quantitative trait loci for resistance to Fusarium head blight in a Chinese wheat landrace Haiyanzhong. Theoretical and Applied Genetics, 122, 1497–1502. 10.1007/s00122-011-1549-0 21344182

[tpg270073-bib-0057] Lin, F. , Xue, S. L. , Zhang, Z. Z. , Zhang, C. Q. , Kong, Z. X. , Yao, G. Q. , Tian, D. G. , Zhu, H. L. , Li, C. J. , Cao, Y. , Wei, J. B. , Luo, Q. Y. , & Ma, Z. Q. (2006). Mapping QTL associated with resistance to Fusarium head blight in the Nanda2419 × Wangshuibai population. II: Type I resistance. Theoretical and Applied Genetics, 112, 528–535. 10.1007/s00122-005-0156-3 16328229

[tpg270073-bib-0058] Liu, S. , Christopher, M. D. , Griffey, C. A. , Hall, M. D. , Gundrum, P. G. , & Brooks, W. S. (2012). Molecular characterization of resistance to Fusarium head blight in US soft red winter wheat breeding line VA00W‐38. Crop Science, 52, 2283–2292. 10.2135/cropsci2012.03.0144

[tpg270073-bib-0059] Liu, S. , Griffey, C. A. , Hall, M. D. , McKendry, A. L. , Chen, J. , Brooks, W. S. , Brown‐Guedira, G. , Van Sanford, D. , & Schmale, D. G. (2013). Molecular characterization of field resistance to Fusarium head blight in two US soft red winter wheat cultivars. Theoretical and Applied Genetics, 126, 2485–2498. 10.1007/s00122-013-2149-y 23832049 PMC3782633

[tpg270073-bib-0060] Liu, S. , Hall, M. D. , Griffey, C. A. , & McKendry, A. L. (2009). Meta‐analysis of QTL associated with Fusarium head blight resistance in wheat. Crop Science, 49, 1955–1968. 10.2135/cropsci2009.03.0115

[tpg270073-bib-0061] Lu, Q. , Lillemo, M. , Skinnes, H. , He, X. , Shi, J. , Ji, F. , Dong, Y. , & Bjørnstad, Å. (2013). Anther extrusion and plant height are associated with Type I resistance to Fusarium head blight in bread wheat line ‘Shanghai‐3/Catbird.’ Theoretical and Applied Genetics, 126, 317–334. 10.1007/s00122-012-1981-9 23052019

[tpg270073-bib-0062] Ma, H. X. , Bai, G. H. , Gill, B. S. , & Hart, L. P. (2006). Deletion of a chromosome arm altered wheat resistance to Fusarium head blight and deoxynivalenol accumulation in Chinese Spring. Plant Disease, 90, 1545–1549. 10.1094/PD-90-1545 30780974

[tpg270073-bib-0063] Maccaferri, M. , Harris, N. S. , Twardziok, S. O. , Pasam, P. K. , Gundlach, H. , Spannagl, M. , Ormanbekova, D. , Lux, T. , Prade, V. M. , Milner, S. G. , Himmelbach, A. , Mascher, M. , Bagnaresi, P. , Faccioli, P. , Cozzi, P. , Lauria, M. , Lazzari, B. , Stella, A. , Manconi, A. , … Cattivelli, L. (2019). Durum wheat genome highlights past domestication signatures and future improvement targets. Nature Genetics, 51, 885–895. 10.1038/s41588-019-0381-3 30962619

[tpg270073-bib-0064] Marcussen, T. , Sandve, S. R. , Heier, L. , Spannagl, M. , Pfeifer, M. , Jakobsen, K. S. , Wulff, B. B. H. , Steuernagel, B. , Mayer, K. F. X. , & Praud, S. , International Wheat Genome Sequencing Consortium . (2014). Ancient hybridizations among the ancestral genomes of bread wheat. Science, 345, 1250092. 10.1126/science.1250092 25035499

[tpg270073-bib-0065] McCartney, C. A. , Brûlé‐Babel, A. L. , Fedak, G. , Martin, R. A. , McCallum, B. D. , Gilbert, J. , Hiebert, C. W. , & Pozniak, C. J. (2016). Fusarium head blight resistance QTL in the spring wheat cross Kenyon/86ISMN 2137. Frontiers in Microbiology, 7, 1542. 10.3389/fmicb.2016.01542 27790188 PMC5061752

[tpg270073-bib-0066] Mesterhazy, A. (1995). Types and components of resistance to Fusarium head blight of wheat. Plant Breeding, 114, 377–386. 10.1111/j.1439-0523.1995.tb00816.x

[tpg270073-bib-0067] Miedaner, T. , Reinbrecht, C. , Lauber, U. , Schollenberger, M. , & Geiger, H. H. (2001). Effects of genotype and genotype—environment interaction on deoxynivalenol accumulation and resistance to Fusarium head blight in rye, triticale, and wheat. Plant Breeding, 120, 97–105. 10.1046/j.1439-0523.2001.00580.x

[tpg270073-bib-0068] Mujeeb‐Kazi, A. , Delgado, R. , Juarez, L. , & Cano, S. (2001). Scab resistance (type II: Spread) in synthetic hexaploid germplasm. Annual Wheat Newsletter, 47, 118–120.

[tpg270073-bib-0069] Nirmala, J. , Saini, J. , Newcomb, M. , Olivera, P. , Gale, S. , Klindworth, D. , Elias, E. , Talbert, L. , Chao, S. , Faris, J. , Xu, S. , Jin, Y. , & Rouse, M. N. (2017). Discovery of a novel stem rust resistance allele in durum wheat that exhibits differential reactions to Ug99 isolates. G3 Genes|Genomes|Genetics, 7, 3481–3490. 10.1534/g3.117.300209 28855282 PMC5633396

[tpg270073-bib-0070] Nyquist, W. E. , & Baker, R. J. (1991). Estimation of heritability and prediction of selection response in plant populations. Critical Reviews in Plant Sciences, 10, 235–322. 10.1080/07352689109382313

[tpg270073-bib-0071] Ogbonnaya, F. C. , Abdalla, O. , Mujeeb‐Kazi, A. , Kazi, A. G. , Xu, S. S. , Gosman, N. , Lagudah, E. S. , Bonnett, D. , Sorrells, M. E. , & Tsujimoto, H. (2013). Synthetic hexaploids: Harnessing species of the primary gene pool for wheat improvement. Plant Breeding Reviews, 37, 35–122. 10.1002/9781118497869.ch2

[tpg270073-bib-0072] Oliver, R. E. , Cai, X. , Friesen, T. L. , Halley, S. , Stack, R. W. , & Xu, S. S. (2008). Evaluation of Fusarium head blight resistance in tetraploid wheat (*Triticum turgidum* L.). Crop Science, 48, 213–222. 10.2135/cropsci2007.03.0129

[tpg270073-bib-0073] Oliver, R. E. , Cai, X. , Xu, S. S. , Chen, X. , & Stack, R. W. (2005). Wheat‐alien species derivatives: A novel source of resistance to Fusarium head blight in wheat. Crop Science, 45, 1353–1360. 10.2135/cropsci2004.0503

[tpg270073-bib-0074] Oliver, R. E. , Stack, R. W. , Miller, J. D. , & Cai, X. (2007). Reaction of wild emmer wheat accessions to Fusarium head blight. Crop Science, 47, 893–897. 10.2135/cropsci2006.08.0531

[tpg270073-bib-0075] Otto, C. D. , Kianian, S. F. , Elias, E. M. , Stack, R. W. , & Joppa, L. R. (2002). Genetic dissection of a major Fusarium head blight QTL in tetraploid wheat. Plant Molecular Biology, 48, 625–632. 10.1023/A:1014821929830 11999839

[tpg270073-bib-0076] Parry, D. W. , Jenkinson, P. , & McLeod, L. (1995). Fusarium ear blight (scab) in small grain cereals—A review. Plant Pathology, 44, 207–238. 10.1111/j.1365-3059.1995.tb02773.x

[tpg270073-bib-0077] Pestka, J. (2010). Toxicological mechanisms and potential health effects of deoxynivalenol and nivalenol. World Mycotoxin Journal, 3, 323–347. 10.3920/WMJ2010.1247

[tpg270073-bib-0078] Petersen, S. , Lyerly, J. H. , Maloney, P. V. , Brown‐Guedira, G. , Cowger, C. , Costa, J. M. , Dong, Y. , & Murphy, J. P. (2016). Mapping of Fusarium head blight resistance quantitative trait loci in winter wheat cultivar NC‐Neuse. Crop Science, 56, 1473–1483. 10.2135/cropsci2015.05.0312

[tpg270073-bib-0079] Puri, K. D. , & Zhong, S. (2010). The 3ADON population of *Fusarium graminearum* found in North Dakota is more aggressive and produces a higher level of DON than the prevalent 15ADON population in spring wheat. Phytopathology, 100, 1007–1014. 10.1094/PHYTO-12-09-0332 20839936

[tpg270073-bib-0080] Ren, J. , Wang, Z. , Du, Z. , Che, M. , Zhang, Y. , Quan, W. , Wang, Y. , Jiang, X. , & Zhang, Z. (2019). Detection and validation of a novel major QTL for resistance to Fusarium head blight from *Triticum aestivum* in the terminal region of chromosome 7DL. Theoretical and Applied Genetics, 132, 241–255. 10.1007/s00122-018-3213-4 30327846

[tpg270073-bib-0081] Ruan, Y. , Comeau, A. , Langevin, F. , Hucl, P. , Clarke, J. M. , Brule‐Babel, A. , & Pozniak, C. J. (2012). Identification of novel QTL for resistance to Fusarium head blight in a tetraploid wheat population. Genome, 55, 853–864. 10.1139/gen-2012-0110 23231604

[tpg270073-bib-0082] Rudd, J. C. , Devkota, R. N. , Baker, J. A. , Peterson, G. L. , Lazar, M. D. , Bean, B. , Worrall, D. , Baughman, T. , Marshall, D. , Sutton, R. , Rooney, L. W. , Nelson, L. R. , Fritz, A. K. , Weng, Y. , Morgan, G. D. , & Seabourn, B. W. (2014). ‘TAM 112’ wheat, resistant to greenbug and wheat curl mite and adapted to the dryland production system in the Southern High Plains. Journal of Plant Registrations, 8, 291–297. 10.3198/jpr2014.03.0016crc

[tpg270073-bib-0083] Rudd, J. C. , Devkota, R. N. , Ibrahim, A. M. , Baker, J. A. , Baker, S. , Sutton, R. , Simoneaux, B. , Opena, G. , Hathcoat, D. , Awika, J. M. , Nelson, L. R. , Liu, S. , Xue, Q. , Bean, B. , Neely, C. B. , Duncan, R. W. , Seabourn, B. W. , Bowden, R. L. , Jin, Y. , … Graybosch, R. A. (2019). ‘TAM 204’ wheat, adapted to grazing, grain, and graze‐out production systems in the southern high plains. Journal of Plant Registrations, 13, 377–382. 10.3198/jpr2018.12.0080crc

[tpg270073-bib-0084] Salamini, F. , Özkan, H. , Brandolini, A. , Schäfer‐Pregl, R. , & Martin, W. (2002). Genetics and geography of wild cereal domestication in the near east. Nature Reviews Genetics, 3, 429–441. 10.1038/nrg817 12042770

[tpg270073-bib-0085] Savary, S. , Willocquet, L. , Pethybridge, S. J. , Esker, P. , McRoberts, N. , & Nelson, A. (2019). The global burden of pathogens and pests on major food crops. Nature Ecology & Evolution, 3, 430–439. 10.1038/s41559-018-0793-y 30718852

[tpg270073-bib-0086] Schmolke, M. , Zimmermann, G. , Buerstmayr, H. , Schweizer, G. , Miedaner, T. , Korzun, V. , Ebmeyer, E. , & Hartl, L. (2005). Molecular mapping of Fusarium head blight resistance in the winter wheat population Dream/Lynx. Theoretical and Applied Genetics, 111, 747–756. 10.1007/s00122-005-2060-2 15947905

[tpg270073-bib-0087] Sharma, J. S. , Overlander, M. , Faris, J. D. , Klindworth, D. L. , Rouse, M. N. , Kang, H. , Long, Y. , Jin, Y. , Lagudah, E. S. , & Xu, S. S. (2021). Characterization of synthetic wheat line Largo for resistance to stem rust. G3 Genes|Genomes|Genetics, 11, jkab193. 10.1093/g3journal/jkab193 34849816 PMC8496286

[tpg270073-bib-0088] Somers, D. J. , Fedak, G. , Clarke, J. , & Cao, W. (2006). Mapping of FHB resistance QTLs in tetraploid wheat. Genome, 49, 1586–1593. 10.1139/g06-127 17426773

[tpg270073-bib-0089] Somers, D. J. , Fedak, G. , & Savard, M. (2003). Molecular mapping of novel genes controlling *Fusarium* head blight resistance and deoxynivalenol accumulation in spring wheat. Genome, 46, 555–564. 10.1139/g03-033 12897863

[tpg270073-bib-0090] Stack, R. W. , Elias, E. M. , Fetch, J. M. , Miller, J. D. , & Joppa, L. R. (2002). Fusarium head blight reaction of Langdon durum‐*Triticum dicoccoides* chromosome substitution lines. Crop Science, 42, 637–642. 10.2135/cropsci2002.6370

[tpg270073-bib-0091] Stack, R. W. , & McMullen, M. P. (1998). A visual scale to estimate severity of Fusarium head blight in wheat (NDSU PP‐1095). NDSU Extension Service.

[tpg270073-bib-0092] Su, Z. , Bernardo, A. , Tian, B. , Chen, H. , Wang, S. , Ma, H. , Cai, S. , Liu, D. , Zhang, D. , Li, T. , Trick, H. , Amand, P. S. , Yu, J. , Zhang, Z. , & Bai, G. (2019). A deletion mutation in *TaHRC* confers *Fhb1* resistance to Fusarium head blight in wheat. Nature Genetics, 51, 1099–1105. 10.1038/s41588-019-0425-8 31182809

[tpg270073-bib-0093] Szabo‐Hever, A. , Zhang, Q. , Friesen, T. L. , Zhong, S. , Elias, E. M. , Cai, X. , Jin, Y. , Faris, J. D. , Chao, S. , & Xu, S. S. (2018). Genetic diversity and resistance to Fusarium head blight in synthetic hexaploid wheat derived from *Aegilops tauschii* and diverse *Triticum turgidum* subspecies. Frontiers in Plant Science, 9, 1829. 10.3389/fpls.2018.01829 30619402 PMC6298526

[tpg270073-bib-0094] Thambugala, D. , Brûlé‐Babel, A. L. , Blackwell, B. A. , Fedak, G. , Foster, A. J. , MacEachern, D. , Gilbert, J. , Henriquez, M. A. , Martin, R. A. , McCallum, B. D. , Spaner, D. , Iqbal, M. , Pozniak, C. J. , N'Diaye, A. , & McCartney, C. A. (2020). Genetic analyses of native Fusarium head blight resistance in two spring wheat populations identifies QTL near the *B1, Ppd‐D1, Rht‐1, Vrn‐1, Fhb1, Fhb2*, and *Fhb5* loci. Theoretical and Applied Genetics, 133, 2775–2796. 10.1007/s00122-020-03631-y 32556394

[tpg270073-bib-0095] Venske, E. , Dos Santos, R. S. , Farias, D. D. R. , Rother, V. , Da Maia, L. C. , Pegoraro, C. , & Costa de Oliveira, A. (2019). Meta‐analysis of the QTLome of Fusarium head blight resistance in bread wheat: Refining the current puzzle. Frontiers in Plant Science, 10, 727. 10.3389/fpls.2019.00727 31263469 PMC6585393

[tpg270073-bib-0096] Viviani, A. , Haile, J. K. , Fernando, W. G. D. , Ceoloni, C. , Kuzmanović, L. , Lhamo, D. , Gu, Y.‐Q. , Xu, S. S. , Cai, X. , Buerstmayr, H. , Elias, E. M. , Confortini, A. , Bozzoli, M. , Brar, G. S. , Ruan, Y. , Berraies, S. , Hamada, W. , Oufensou, S. , Jayawardana, M. , … Cattivelli, L. (2025). Priority actions for Fusarium head blight resistance in durum wheat: Insights from the wheat initiative. The Plant Genome, 18, e20539. 10.1002/tpg2.20539 39757924 PMC11701714

[tpg270073-bib-0097] Wang, H. , Sun, S. , Ge, W. , Zhao, L. , Hou, B. , Wang, K. , Lyu, Z. , Chen, L. , Xu, S. , Guo, J. , Li, M. , Su, P. , Li, X. , Wang, G. , Bo, C. , Fang, X. , Zhuang, W. , Cheng, X. , Wu, J. , … Kong, L. (2020). Horizontal gene transfer of *Fhb7* from fungus underlies Fusarium head blight resistance in wheat. Science, 368, eaba5435. 10.1126/science.aba5435 32273397

[tpg270073-bib-0098] Wang, R. , Chen, J. , Anderson, J. A. , Zhang, J. , Zhao, W. , Wheeler, J. , Klassen, N. , See, D. R. , & Dong, Y. (2017). Genome‐wide association mapping of Fusarium head blight resistance in spring wheat lines developed in the Pacific Northwest and CIMMYT. Phytopathology, 107, 1486–1495. 10.1094/PHYTO-02-17-0073-R 28703042

[tpg270073-bib-0099] Wang, X. , Li, G. , Jia, H. , Cheng, R. , Zhong, J. , Shi, J. , Chen, R. , Wen, Y. , & Ma, Z. (2024). Breeding evaluation and precise mapping of*Fhb8* for Fusarium head blight resistance in wheat (*Triticum aestivum*). Plant Breeding, 143(1), 26–33. 10.1111/pbr.13113

[tpg270073-bib-0100] Wulff, B. B. , & Jones, J. D. (2020). Breeding a fungal gene into wheat. Science, 368, 822–823. 10.1126/science.abb9991 32439778

[tpg270073-bib-0101] Xu, S. S. , Khan, K. , Klindworth, D. L. , & Nygard, G. (2010). Evaluation and characterization of high‐molecular weight 1D glutenin subunits from *Aegilops tauschii* in synthetic hexaploid wheats. Journal of Cereal Science, 52, 333–336. 10.1016/j.jcs.2010.05.004

[tpg270073-bib-0102] Yan, H. , Li, G. , Shi, J. , Tian, S. , Zhang, X. , Cheng, R. , Wang, X. , Yuan, Y. , Cao, S. , Zhou, J. , Kong, Z. , Jia, H. , & Ma, Z. (2021). Genetic control of Fusarium head blight resistance in two Yangmai 158‐derived recombinant inbred line populations. Theoretical and Applied Genetics, 134, 3037–3049. 10.1007/s00122-021-03876-1 34110431

[tpg270073-bib-0103] Yang, J. , Hu, C. C. , Ye, X. Z. , & Zhu, J. (2005). QTLNetwork 2.0. Institute of Bioinformatics, Zhejiang University. http://ibi.zju.edu.cn/software/qtlnetwork

[tpg270073-bib-0104] Yang, Z. , Gilbert, J. , Fedak, G. , & Somers, D. J. (2005). Genetic characterization of QTL associated with resistance to Fusarium head blight in a doubled‐haploid spring wheat population. Genome, 48, 187–196. 10.1139/g04-104 15838540

[tpg270073-bib-0105] Yi, X. , Cheng, J. , Jiang, Z. , Hu, W. , Bie, T. , Gao, D. , Li, D. , Wu, R. , Li, Y. , Chen, S. , Cheng, X. , Liu, J. , Zhang, Y. , & Cheng, S. (2018). Genetic analysis of Fusarium head blight resistance in CIMMYT bread wheat line C615 using traditional and conditional QTL mapping. Frontiers in Plant Science, 9, 573. 10.3389/fpls.2018.00573 29780395 PMC5946024

[tpg270073-bib-0106] Zhang, F. , Zhang, H. , Liu, J. , Ren, X. , Ding, Y. , Sun, F. , Zhu, Z. , He, X. , Zhou, Y. , Bai, G. , Ni, Z. , Sun, Q. , & Su, Z. (2024). *Fhb9*, a major QTL for Fusarium head blight resistance improvement in wheat. Journal of Integrative Agriculture. 10.1016/j.jia.2024.03.045

[tpg270073-bib-0107] Zhang, L. , Liu, D. , Lan, X. , Zhang, Y. , & Yan, Z. (2008). A synthetic wheat with 56 chromosomes derived from *Triticum turgidum* and *Aegilops tauschii* . Journal of Applied Genetics, 49, 41–44. 10.1007/BF03195247 18263968

[tpg270073-bib-0108] Zhang, Q. , Axtman, J. E. , Faris, J. D. , Chao, S. , Zhang, Z. , Friesen, T. L. , Zhong, S. , Cai, X. , Elias, E. M. , & Xu, S. S. (2014). Identification and molecular mapping of quantitative trait loci for Fusarium head blight resistance in emmer and durum wheat using a single nucleotide polymorphism‐based linkage map. Molecular Breeding, 34, 1677–1687. 10.1007/s11032-014-0180-6

[tpg270073-bib-0109] Zhao, M. , Leng, Y. , Chao, S. , Xu, S. S. , & Zhong, S. (2018). Molecular mapping of QTL for Fusarium head blight resistance introgressed into durum wheat. Theoretical and Applied Genetics, 131, 1939–1951. 10.1007/s00122-018-3124-4 29869075

[tpg270073-bib-0110] Zheng, T. , Hua, C. , Li, L. , Sun, Z. , Yuan, M. , Bai, G. , Humphreys, G. , & Li, T. (2021). Integration of meta‐QTL discovery with omics: Towards a molecular breeding platform for improving wheat resistance to Fusarium head blight. The Crop Journal, 9, 739–749. 10.1016/j.cj.2020.10.006

[tpg270073-bib-0111] Zhu, Z. , Bonnett, D. , Ellis, M. , He, X. , Heslot, N. , Dreisigacker, S. , Gao, C. , & Singh, P. (2016). Characterization of Fusarium head blight resistance in a CIMMYT synthetic‐derived bread wheat line. Euphytica, 208, 367–375. 10.1007/s10681-015-1612-z

