# Supplementary material for: Identification and mapping of quantitative trait loci for Fusarium head blight resistance in a synthetic hexaploid × hard red spring wheat population
Source: Plant Genome. 2025 Jul 9;18(3):e70073. doi: 10.1002/tpg2.70073 (PMC12241835; doi:10.1002/tpg2.70073)
Supplement: Supplementary file 1 — Supplemental Table S1. Whole genome linkage map for the ND495 × Largo recombinant inbred line population. Supplemental Table S2. Genotypic data for ND495 × Largo population of 188 recombinant inbred lines genotyped with the wheat Infinium 90K SNP iSelect array and SSR markers. Supplemental Table S3. Least significant difference (LSD) tests for the Fusarium head blight (FHB) severity data of the ND495 × Largo recombinant inbred lines, their parents, and the resistant and susceptible checks (Sumai 3 and Wheaton, respectively) from the greenhouse experiment in 2018 (18GH). Supplemental Table S4. Least significant difference (LSD) tests for the Fusarium head blight (FHB) severity data of the ND495 × Largo recombinant inbred lines, their parents, and the resistant and susceptible checks (Sumai 3 and Wheaton, respectively) from the greenhouse experiment in 2019 (19GH). Supplemental Table S5. Least significant difference (LSD) tests for the Fusarium head blight (FHB) severity data of the ND495 × Largo recombinant inbred lines, their parents, and the resistant and susceptible checks (Sumai 3 and Wheaton, respectively) from the field experiment in 2018 (18F). Supplemental Table S6. Least significant difference (LSD) tests for the Fusarium head blight (FHB) severity data of the ND495 × Largo recombinant inbred lines, their parents, and the resistant and susceptible checks (Sumai 3 and Wheaton, respectively) from the field experiment in 2019 (19F). Supplemental Table S7. Number of markers and length for each chromosome of the ND495/Largo RIL population. Supplemental Table S8. List of candidate genes in the QFhb.rwg‐1D region on chromosome 1D. Supplemental Table S9. List of candidate genes in the QFhb.rwg‐2D region on chromosome 2D. Supplemental Table S10. List of candidate genes in the QFhb.rwg‐7D.2 region on chromosome 7D. Supplemental Table S11. Comparison of allele information derived from significant SNP markers and their corresponding KASP markers. Supplemental Table S12. [file TPG2-18-e70073-s001.docx]

Supplemental material

**Supplemental Table S1**. Whole genome linkage map for the ND495 × Largo recombinant inbred line population.

**Supplemental Table S2**. Genotypic data for ND495 × Largo population of 188 recombinant inbred lines genotyped with the wheat Infinium 90K SNP iSelect array and SSR markers.

**Supplemental Table S3**. Least significant difference (LSD) tests for the Fusarium head blight (FHB) severity data of the ND495 × Largo recombinant inbred lines, their parents, and the resistant and susceptible checks (Sumai 3 and Wheaton, respectively) from the greenhouse experiment in 2018 (18GH).

**Supplemental Table S4**. Least significant difference (LSD) tests for the Fusarium head blight (FHB) severity data of the ND495 × Largo recombinant inbred lines, their parents, and the resistant and susceptible checks (Sumai 3 and Wheaton, respectively) from the greenhouse experiment in 2019 (19GH).

**Supplemental Table S5**. Least significant difference (LSD) tests for the Fusarium head blight (FHB) severity data of the ND495 × Largo recombinant inbred lines, their parents, and the resistant and susceptible checks (Sumai 3 and Wheaton, respectively) from the field experiment in 2018 (18F).

**Supplemental Table S6**. Least significant difference (LSD) tests for the Fusarium head blight (FHB) severity data of the ND495 × Largo recombinant inbred lines, their parents, and the resistant and susceptible checks (Sumai 3 and Wheaton, respectively) from the field experiment in 2019 (19F).

**Supplemental Table S7**. Number of markers and length for each chromosome of the ND495/Largo RIL population.

**Supplemental Table S8**. List of candidate genes in the *QFhb.rwg-1D* region on chromosome 1D.

**Supplemental Table S9**. List of candidate genes in the *QFhb.rwg-2D* region on chromosome 2D.

**Supplemental Table S10**. List of candidate genes in the *QFhb.rwg-7D.2* region on chromosome 7D.

**Supplemental Table S11**. Comparison of allele information derived from significant SNP markers and their corresponding KASP markers.

**Supplemental Table S12**. Allele information of KASP markers on the 25 resistant SHW lines published by [Szabo-Hever et al. (2018)](#bib93). For comparison, the resistant allele, susceptible allele, ND495, and Largo genotype information from the current study are listed.

**Supplemental Figure S1**. Box plot diagrams of FHB severities (%) of the QTL groups of the ND495/Largo RILs in each of the environments. 18GH, 19GH, 18F, and 19F are FHB severity data from greenhouse and field experiments in 2018 and 2019, respectively. FHBALL are average FHB severity data from 18F, 19F, 18GH, and 19GH experiments. The means with the same letters on the top of the whiskers did not differ significantly (α < 0.05) as determined by LSD.

**Supplemental Figure S2**. Validation of KASP markers linked to FHB resistance QTL *QFhb.rwg-1D*, *QFhb.rwg-2D*, and *QFhb.rwg-5B* in the ND495/Largo RIL population and the 25 resistant SHW lines published by [Szabo-Hever et al. (2018)](#bib93). The Letters “R” and “S” represent resistant and susceptible alleles, respectively.
